# Triage tools to inform the prioritisation of physical health services following a diagnosis of cancer: a scoping review

**DOI:** 10.1007/s00520-025-09816-9

**Published:** 2025-08-06

**Authors:** Georgia L. White, Lauren C. Capozzi, Corey Linton, Adrian Wright, Tamara Jones, Hattie H. Wright, Kate A. Bolam, Elizabeth A. Johnston, Briana K. Clifford, Keegan Bean, Stephanie Brown, Sarah Kolesaric, Mary A. Kennedy, Bryan A. Chan, Grace L. Rose

**Affiliations:** 1 School of Health, University of the Sunshine Coast, Sippy Downs, Queensland Australia; 2 Department of Clinical Neurosciences, Cumming School of Medicine, University of Calgary, Calgary, Alberta Canada; 3 School of Psychological Sciences, University of Melbourne, Parkville, Victoria Australia; 4 Person-Centred Research, Eastern Health Clinical School, Faculty of Medicine, Nursing and Health Sciences, Monash University, Clayton, Victoria Australia; 5 Sunshine Coast Health Institute, Birtinya, Queensland Australia; 6 Cardiometabolic Health and Exercise Physiology Laboratory, Baker Heart and Diabetes Institute, Melbourne, Victoria Australia; 7 Department of Physical Activity and Health, The Swedish School of Sport and Health Sciences, Stockholm, Sweden; 8 Viertel Cancer Research Centre, Cancer Council Queensland, Brisbane, Queensland Australia; 9 School of Exercise and Nutrition Sciences, Faculty of Health, Queensland University of Technology, Brisbane, Queensland Australia; 10 School of Health Sciences, The University of New South Wales, Sydney, New South Wales Australia; 11 Nutrition and Health Innovation Research Institute, Edith Cowan University, Joondalup, Western Australia Australia; 12 Sunshine Coast University Hospital, Birtinya, Queensland Australia

**Keywords:** Health services, Oncology, Physical rehabilitation, Needs assessment, Supportive care

## Abstract

**Purpose:**

Many people face multiple cancer- and treatment-related sequalae. Triage and referral to physical health services can manage such consequences, but a comprehensive understanding of available triage tools is lacking. This review (i) identifies tools used to triage to physical health services, (ii) maps tool characteristics and application outcomes and (iii) summarises existing gaps.

**Methods:**

A systematic search was conducted (three databases, April 2024). Articles were included if they used a tool to triage to physical health services. Tools were classified by triaged disciplines (i.e., diet, exercise, physical rehabilitation, multidisciplinary) and screened physical impairments (e.g., malnutrition). Tool characteristics (e.g., triage method) and application outcomes (i.e., reach, triage rates) were extracted.

**Results:**

Of 23,369 records retrieved, 67 studies were included. Studies comprised 78 instances of tool use (64 unique tools), where *n* = 33 triaged to dietetics (42%), *n* = 6 exercise (8%), *n* = 11 physical rehabilitation (14%), and *n* = 28 a combination of health disciplines (36%). Mean age was 65 years. Most tools were used during-treatment (45%), in hospital settings (62%), measured malnutrition/physical function (60%) and used single cut-off scores (68%). Reach and triage rates varied, with exercise (reach = 89%) and diet (triage = 63%) rates highest.

**Conclusion:**

Many physical health triage tools exist, most solely for dietetics, with heterogeneous characteristics and application outcomes. Updated tools are needed for triage to exercise/physical rehabilitation, multiple age cohorts across the cancer continuum, and that potentially use multiple cut-off scores. Cancer care professionals can use this compendium to identify which tool characteristics best suit their healthcare setting, for optimal outcomes.

**Supplementary Information:**

The online version contains supplementary material available at 10.1007/s00520-025-09816-9.

## Introduction

Globally, the number of people diagnosed with cancer continues to increase; in 2022, there were approximately 20 million new cancer diagnoses; this number is expected to increase to 35 million in the next 25 years [1]. Access and referral to health services is essential to improve the outcomes of people post-diagnosis and -treatment [2, 3]. In some countries such as Australia, Canada, and the United States, secondary and tertiary health services include, but are not limited to, psychology, social work, exercise, rehabilitation (e.g., occupational, physical therapy, cancer physiatry, speech therapy), and dietetic services [2]. Such services are essential to both the physical and psychosocial wellbeing of people following a cancer diagnosis [2, 4]. Indeed, screening and early triage for, referral to, and coordination with allied health services is desired by people diagnosed with cancer [5, 6] and aligns with best practice recommendations for cancer care [4, 7–10].

Despite most Australian healthcare systems providing supportive care services [11], several challenges limit their effectiveness. One barrier to equitable service access is the lack of standardised pathways to intervention referral [12, 13]. This ultimately limits service provision to address survivorship and quality of life among people living with, or beyond cancer. Indeed, a key challenge to the provision of effective health service delivery is consistent and clear identification of patients who would benefit most from physical health support, and the type of intervention that would best suit their individual needs. This challenge can be addressed in practice through the consistent use of screening and triaging tools, or needs assessments, that comprise of singular or multiple composite assessments to ultimately inform the need for service [14]. Such tools could enable healthcare services to optimise resource allocation, including the type and timing of allied health support that an individual may need. It also ensures equitable identification of those in need of services, as opposed to relying on busy care providers to identify needs ad hoc.

Unmet supportive care needs continue to be reported by people following a cancer diagnosis, and include a lack of access to health information [6, 15] as well as physical impairments or concerns such as fatigue, pain, and weight loss [5]. Though a variety of exercise, rehabilitation, and dietary triage tools exist [16–18], the continued challenge of high rates of unmet needs of people following a cancer diagnosis highlights the importance of optimising current supportive care screening, triage, and referral to address physical needs. Understanding tool characteristics and application outcomes can provide insight into factors that influence the success of triage tools in healthcare settings [19]. To date there has been no comprehensive understanding of which tools have been used or evaluated for the purpose of triage to physical health services in oncology practice, or to systematically evaluate their application outcomes or possible gaps in existing tools. Therefore, this systematic scoping review aimed to identify:

1. Available tools and tool characteristics (e.g., setting, measures included, method and outcome of triage) used among people following a cancer diagnosis, for the purpose of triage or prioritisation to receive exercise, physical rehabilitation, and/or dietary intervention.
2. Application outcomes of included physical health triage tools (e.g., reach, acceptability).
3. Limitations of existing physical health triage tools.

## Methods

### Search strategy

This systematic scoping review was conducted in accordance with the PRISMA Extension for Scoping Reviews [20], and was pre-registered with Open Science Framework (ID: osf.io/z2j69/). Searches were conducted up to 24th April 2024 across three databases (PubMed, CINAHL, Scopus). The search included combinations of relevant free-text terms amalgamated with Boolean operators, searched in title/abstract/keyword and limited to articles published after 1995, excluding key words for animal models in title/abstract (i.e., mice, rat, animal). Global search terms for population, triage tool, and relevant allied health were included in ‘and’ combination, with synonyms included in ‘or’ combination (Supplementary Table 1a)*.*

### Selection criteria

The inclusion criteria were: (i) Design: randomised controlled trials (RCT), quasi-RCTs, cohort studies, pre-post single arm studies, cross-sectional studies. (ii) Population: adults (aged ≥ 18 years) post-cancer diagnosis. (iii) Intervention: electronic or physical ‘tool’ that measures physical needs and has been used or applied for the purpose of patient triage, prioritisation, or referral to exercise, physical activity, diet, or physical rehabilitative care. Physical health tool(s) that were combined with, or within, a non-physical health tool (e.g., medicine, nursing, psychological or distress) were included, with data extracted for the physical health component only. The exclusion criteria were (i) no full text available, (ii) published in languages other than English, (iii) incorrect publication type, (iv) data of interest specific to triage tools could not be adequately extracted. Full details of the review inclusion and exclusion criteria can be found in Supplementary Table 1b.

### Article appraisal

All articles underwent automatic de-duplication (Covidence, Veritas Health, Australia) followed by manual de-duplication. Titles and abstracts were screened by two independent reviewers, including A.W, S.B, G.W, G.R, C.L, E.J, K.B, T.J, K.A.B, L.C, H.W. Following initial title and abstract exclusion, full text articles were assessed against the full inclusion and exclusion criteria by two independent reviewers, including G.R, G.W, T.J, K.B, C.L, B.C, H.W, A.W, E.A.J, L.C, K.A.B, S.K, S.B. Discrepancies were resolved by G.R. Descendancy and ascendancy approaches were used to identify additional eligible studies, namely the reference lists and citing articles (as per Scopus) of included studies, respectively. Corresponding authors of eligible studies were contacted in the instance that full text was not available and details for correspondence could be found (*n* = 3; *n* = 1 supplied full text) or further information was required (*n* = 22).

### Data extraction and synthesis

Data extraction was completed using a pre-defined data extraction spreadsheet and included details regarding study information, participant characteristics, tool and screening system details, and application outcomes. Application outcomes including reach, triage rates, effectiveness (impacts on health-related quality of life), and acceptability were extracted, however only triage rates, acceptability, and reach outcomes were present across the majority of studies and thus reported within this review. Only 13 studies reported acceptability (19%) [16, 21–32] (*n* = 10 studies quantitative, *n* = 3 studies qualitative) (Supplementary Tables 2, 3, 4, 5); as such, whilst this information is reported, it was not synthesised. Data was extracted by 10 reviewers (A.W, B.C, C.L, E.J, G.W, G.R, H.W, L.C, E.A.J, T.J), with 25% cross-check of articles and pre-extraction correction through feedback (G.R). Discrepancies among extractors were < 10% prior to corrections. In studies where multiple discrete tools were used to triage to multiple disciplines, each tool was considered separately based on its discipline. Single tool(s) that triaged to multiple disciplines, were considered a multidisciplinary tool. During data synthesis, several definitions were operationalised for this review (Table 1).

**Table 1:** Definitions applied in review

Term	Definition as applied in this review
Acceptability	The perception among stakeholder (e.g., patients, clinicians) that the tool is appropriate or satisfactory
Exercise	Any form of planned, structured, and repetitive bodily movement performed to improve or maintain fitness or health
Multidisciplinary	Includes at least two physical health disciplines (e.g., dietetics/nutrition and/or exercise physiology, and/or physiotherapy, and/or occupational therapy, and/or speech therapy)
Physical rehabilitation	An intervention focussed on improving function and reducing disability, derived from a singular discipline (i.e., occupational therapy or physiotherapy or speech therapy or physiatry)
Reach	The proportion of target group who used the tool
Tool	An assessment or collection of assessments used to lead to referral (or similar) to a specific discipline – often including a cut-off score or tick box
Triage rate	The number of people triaged (e.g., referral, specific prescription, discussion) compared to the number of people needing to be referred, identified from a “positive” screening Where this was unable to be calculated from the study, “T1” was applied – the number of people referred compared to the number of total people screened using the tool

### Methodological quality assessment

The quality of included articles was assessed by two independent reviewers, including A.W, B.C, C.L, E.J, G.W, G.R, H.W, L.C, E.A.J, T.J, with consensus reached through discussion or a third arbiter (G.R or G.W). All included articles were appraised using a modified Mixed Methods Appraisal Tool (MMAT), as detailed by Hong et al. [33], to enable quality comparison among the multiple study types of articles included within this review. Scoring of the MMAT was calculated based on the number of quality criteria met (i.e., yes response). All identified studies were included in the review regardless of quality rating.

## Results

### Search and selection of studies

**Fig. 1:**
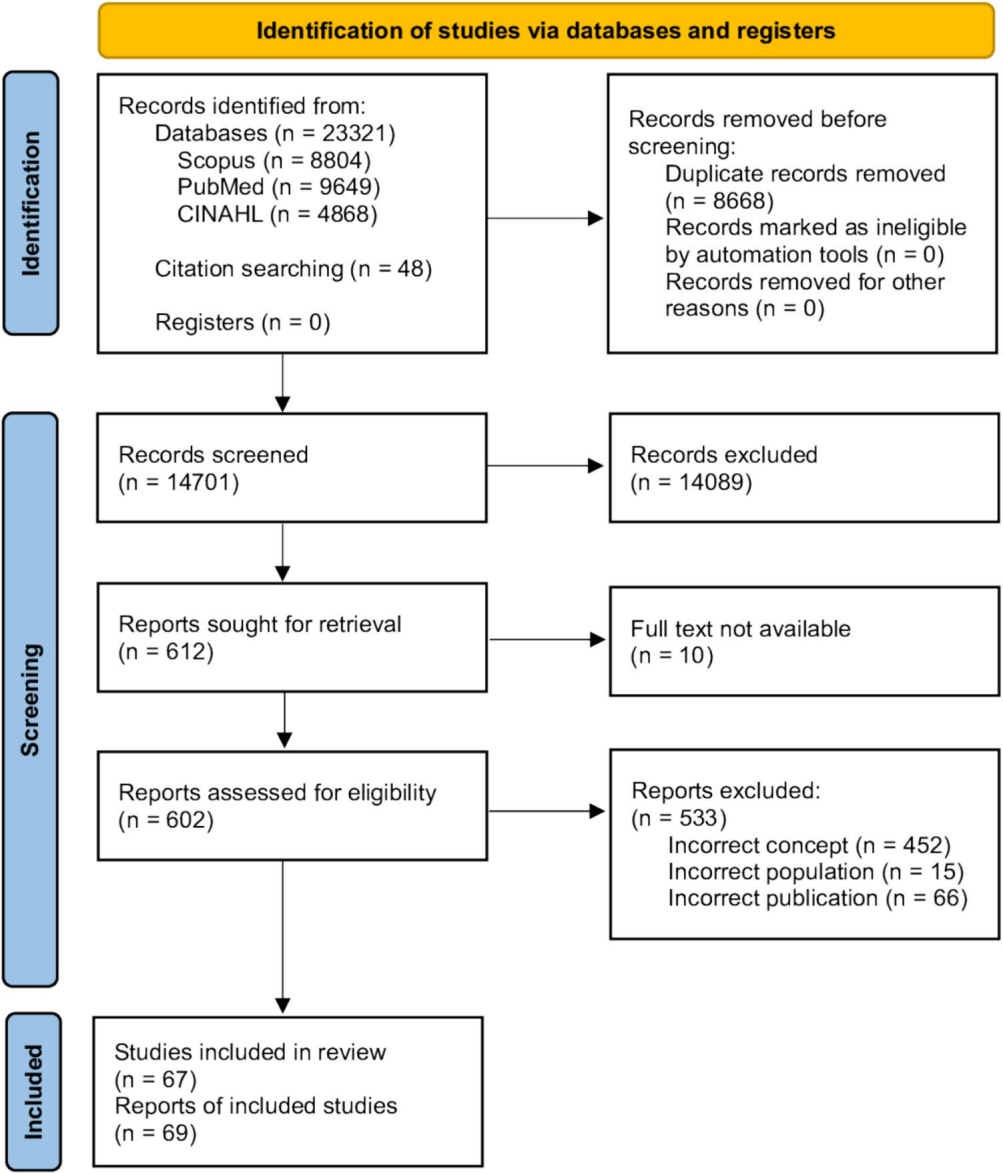
PRISMA diagram

The systematic search resulted in a total of 23,369 records, including 48 records identified through forwards and backwards citation searches. Following the removal of duplicates, the abstracts of 14,701 records were screened. The full text of 602 articles were independently reviewed for eligibility, with 69 publications, across 67 studies included in the review [16, 21–32, 34–89] (Fig. 1).

### Study and participant characteristics

Study and participant characteristics for dietetic, exercise, physical rehabilitation, and multidisciplinary studies are shown in Supplementary Tables 2, 3, 4, 5, respectively. Overall, half of the studies were interventional (*n* = 37, 54%), which included 10 randomised controlled trials. Nearly all reports were quantitative (*n* = 64, 93%) and the remainder employed mixed methods (*n* = 5, 7%) (Supplementary Table 6)*.* Less than a quarter of reports (*n* = 14, 20%) included triage or referral rates as their primary outcome. Sample sizes ranged from 11 to 68,119, with a median of 187 (total sample *n* = 99,791), with 56% of participants being female within studies that reported sex distribution of their sample. Whilst many studies explored mixed cancer types (*n* = 31, 46%), nine studies did not report cancer type (13%). On average, participants were aged 65 years (range = 51–81 years; Supplementary Tables 2, 3, 4, 5). Most studies were conducted in a hospital setting (*n* = 41, 61%), cancer centres (*n* = 23, 34%), community cancer care organisations (*n* = 2, 3%) or universities (*n* = 1, 1%).

### Tool characteristics

Across the 67 included studies, a total of 64 unique tools were identified with tools used across 78 separate instances. Henceforth, results are reported relative to total of instances of tool use, due to differing outcomes across identical tools. Most tools led to triage to a singular discipline or singular purpose (*n* = 50, 64%) and the remainder triaged to multidisciplinary physical health services (*n* = 28, 36%). One quarter of the tools used were authors’ original tools (*n* = 20, 26%). Over half of the tools resulted in referral to a specific discipline(s) (*n* = 45, 58%), with fewer tools resulting in a specific prescription (*n* = 11, 14%), discussion (*n* = 1, 1%), notification messages or flags (*n* = 2, 3%), or a combination of outcomes (*n* = 19, 24%). Over a half of tools (*n* = 53, 68%) used a single cut-off score to triage to services. Nearly all studies delivered tools in-person (*n* = 47, 70%), with the remainder delivered online through apps or electronic medical records (*n* = 7, 10%), a combination of in-person and online (*n* = 7, 10%), phone call or mail (*n* = 4, 6%), or not reported (*n* = 2, 3%).

Many tools were used to triage solely to dietetic services (*n* = 33, 42%), including 19 unique tools. Tools mostly assessed malnutrition risk (*n* = 27, 82%), followed by cachexia, and a combination of perceived nutrition needs/symptoms to inform triage (Table 2). The most frequently used tool within the dietetic discipline was the Nutritional Risk Screening 2002 (*n* = 7, 21%) [18]. Limited tools (*n* = 2, 6%) considered referral or screening for weight management (i.e., overnutrition). Few tools were purposed to triage to exercise services (*n* = 6, 8%); these tools screened for physical inactivity, isolated physical outcomes, such as physical function (gait and handgrip strength) and cardiovascular function (6 Minute Walk Test, Cardiopulmonary Exercise Test), or a combination of impairments to inform triage (Table 2). Eleven (14%) tools were used to triage solely to physical rehabilitation services (Table 2), including physiotherapy (*n* = 5, 46%), occupational therapy (*n* = 4, 37%), and speech therapy (*n* = 2, 18%; Supplementary Table 4). Tools that referred to physical rehabilitation services frequently assessed singular impairments, such as activities of daily living and fatigue (*n* = 8, 73%). Most of the multidisciplinary tools (*n* = 21, 75%) assessed multiple impairments. On average, these multidisciplinary tools referred to three services (range = 2–4 services), most commonly dietetics, physiotherapy, and occupational therapy (Supplementary Tables 4 and 7). Only two multidisciplinary tools [23, 74] and one exercise tool [38] included triage to cancer physiatry (Supplementary Table 7).

**Table 2:** Overview of included tools by discipline and impairment

Discipline by impairment	Frequency of impairment screened	Publications	Overview				Outcomes	
	n^1^ (%)	n (publication no)	Application across cancer continuum *n* (%)^2^	Time to complete (mins) range	Cancer type	Triaged health professional	Reach mean % (range)	Triage rate^3^ mean % (range)
**DIETETICS (*n*** **= 33, 42%)**								
*Malnutrition risk*	27 (35)	29 (1,3,10,11,12,13,15,18,20,21,30,32,33,34,36,37,40,44,48,49,51,52,53,59,60,61,62,64,68)	PrT: 5 (19) DT: 16 (59) U: 2 (7) NR: 4 (15)	2–30	Mixed	D, N	56 (5–100)	59 (11–105)*
*Cachexia*	1 (1)	1 (5)	DT: 1 (100)	NR	Lung	D	81 (NA)	67 (NA)
*Combination*	5 (6)	5 (2,22,50,66,69)	PrT: 1 (20) DT: 2 (40) NR: 2 (40)	2–40	Mixed	D, N	69 (52–88)	62 (37–86)
**EXERCISE (*n*** **= 6, 8%)**								
*Physical inactivity*	1 (1)	1 (7)	NR: 1 (100)	NR	Mixed	Ph	100 (NA)	31 (NA)
*Physical function*	1 (1)	1 (40)	DT: 1 (100)	NR	Colorectal	PT	NR	79 (NA)^
*Cardiovascular capacity*	1 (1)	1 (61)	DT: 1 (100)	NR	Mixed	NR	NR	80 (NA)
*Combination*	3 (4)	3 (14,16,56)	DT: 1 (33) PoT: 1 (33) U: 1 (33)	NR	Mixed	PT, OT, Ex NR	78 (NA)	36 (23–53)*^#^
**PHYSICAL REHABILITATION (*n*** **= 11, 14%)**								
*ADLs and/or IADLs*	4 (5)	4 (4,12,29,40)	DT: 2 (50) U: 2 (50)	5–15	Mixed	OT, PT	68 (50–68)	37 (0–100)
*Physical function*	4 (5)	4 (12,27,58,65)	PrT: 1 (25) DT: 1 (25) U: 2 (50)	3–20	Mixed	OT, PT	91 (NA)	13 (0–49)*
*Falls and/or balance*	1 (1)	1 (49)	PrT: 1 (100)	NR	Head and neck	PT	26 (NA)	22.2 (NA)^
*Eating, chewing, swallowing*	2 (3)	2 (42,45)	PrT: 1 (50) DT: 1 (50)	54	Mixed	ST	100 (NA)	67 (47–87)
**MULTIDISCIPLINARY (*n*** **= 28, 36%)**								
*Physical function*	5 (6)	5 (6,11, 28,38,44)	DT: 3 (60) U: 1 (20) NR: 1 (20)	9–20	Mixed	D, PT, PT assistant, OT, ST	95 (88–100)	D: 52 (NA) PT: 24 (20–28)* OT: 15 (11–20)* ST: NR Ex: 84 (83–85)*
*Sarcopenia*	1 (1)	1 (39)	NR: 1 (100)	15	Mixed	D, PT (Ex focussed)	99 (NA)	D: 50 (NA) Ex: 62 (NA)
*Nutrition*	1 (1)	1 (38)	DT: 1 (100)	NR	Mixed	PT, OT, N	100 (NA)	NR
*Combination*	21 (27)	22 (8,9,17,19,23,24,25,26,31,35,41,43,46,47,52,53,54,55,57,59,63,67)	PrT: 1 (5) DT: 5 (24) PoT: 4 (19) U: 7 (33) NR: 4 (19)	5–45	Mixed	D, OT, PT, ST, Ex specialist, swallowing specialist, Ph	58 (3–96)	D: 48 (30–73)* OT: 29 (0–61)* PT: 34 (2–80)* ST: 9 (5–10)* Ex: 100 (NA) Ph: 21 (10–31)

^1^Number of times a tool has been used to assess an impairment, percentage relative to total number of tools included within the review

^2^Percentage relative to number of publications identified within impairment

^3^ T reported, where not stated

*Combination of T and T1 results reported

^T1 results reported only

^#^Triage rate for additional rehabilitation referrals from Dalzell et al. 2017 = 4.25 (0.5–16)

Percentages may not add to 100% due to rounding

Combination: combination of impairments assessed, D: dietitian, Dietetics: dietetic or nutritional services, DT: during-treatment, Ex: exercise, Exercise: exercise services defined as any form of planned, structured, and repetitive bodily movement performed to improve or maintain fitness or health, Multidisciplinary: a combination of physiotherapy, occupational, speech therapy, exercise, and/or dietetics services, Ph: physiatry, physical rehabilitation: physiotherapy, or occupational therapy, or speech therapy services, PrT: pre-treatment, PoT: post-treatment, PT: physiotherapist: N: nutritionist, NA: not applicable, NR: not reported, Reach: the proportion of people who adopted the tool, ST: speech therapist, T: triage rate (the number of people referred/number of people needing to be referred [identified from a positive screening]), T1: where T is NR – triage rate 1 (number of people referred/number of total people screened), U: universal

The application of the tools across the cancer continuum (i.e., pre-, during-, and post-treatment) varied. Most tools were used during treatment (*n* = 35, 45%), with the remaining tools applied universally (*n* = 15, 19%), pre-treatment (*n* = 10, 13%), and post-treatment (*n* = 10, 6%). Treatment phase for the remainder of tools were not reported (*n* = 13, 17%). Time to complete the tools ranged from 2–45 min, however most studies did not report this outcome (*n* = 58, 74%). Forty-nine tools (63%) were identified during data extraction that included non-physical health aspects or were used in conjunction with non-physical health tools for triage to additional services (e.g., nursing, psychological, medical services). Furthermore, one quarter of all tools (*n* = 20, 26%) were extracted from a lengthy battery of assessments [31, 32, 41, 42, 63, 64, 67, 71, 79, 81].

### Tool application outcomes

Reach varied among each triaged discipline (Table 2). Overall, the exercise tools had the highest reach (89%, [mean range: 78–100%], *n* = 2 studies), followed by tools for multidisciplinary services (mean 86% [mean range: 58–100%], *n* = 18 studies), physical rehabilitation (71.3% [26–100%], *n* = 6 studies), and dietetics (69% [55.9–81%], *n* = 24 studies). Considering impairment subcategories within the dietetics discipline, a cachexia screening tool had the highest reach (81%, *n* = 1 study), followed by combination tools (70%, *n* = 2 studies) and screening tools for malnutrition risk (56%, *n* = 19 studies). Reach was only reported in two studies within the exercise discipline, specifically screening physical inactivity (100%) [38] and combination impairments (78%) [76]. The average reach across the physical rehabilitation impairments was 100% for eating, chewing, and swallowing (*n* = 2 studies), 91% for physical function (*n* = 1 study), 68% for (instrumental) activities of daily living (*n* = 2 studies), and 26% for falls/balance (*n* = 1 study). For multidisciplinary services, the average reach for nutrition was 100% (*n* = 1 study), 100% for sarcopenia (*n* = 1 study), 95% for physical function (*n* = 3 studies), and 71% for combination (*n* = 13 studies). Diet tools had the highest triage rate (mean 63% [mean range: 59–67%], *n* = 25 studies), followed by exercise tools (57% [31–80%], *n* = 6 studies), multidisciplinary services (42% [9–100], *n* = 14 studies), and physical rehabilitation (35% [13–67%], *n* = 10 studies).

### Quality assessment

The quality of the included reports within this review varied (Supplementary Table 6)*.* Most studies scored 60% (*n* = 23) or 80% (*n* = 21), meaning 3 or 4 of the 5 criteria for quality were met, respectively. The remaining studies scored 20% (*n* = 3), 40% (*n* = 10), and 100% (*n* = 12). Amongst the included reports, the MMAT criteria that performed the best were criterion 1, 3, and 5. Across differing study designs, criterion 1 addressed randomisation and representation of participants, sampling strategy, or rationale for study design, whilst criterion 3 focussed on measurements and outcomes. Criterion 5, depending on study design, addressed adherence and administration of the intervention, statistical analysis, or quality of both quantitative and qualitative components for mixed methods studies. Criterion 4 had the lowest quality rating, which addressed blinding, confounders, nonresponse bias or cohesiveness between qualitative and quantitative components.

## Discussion

This systematic scoping review synthesised tools that have been used to triage to physical health services for people living with or beyond cancer, identifying 78 instances of tool use (64 unique tools), reported across 67 studies. Nearly half of the tools triaged solely to dietetic services, with few tools available for triage to exercise or physical rehabilitation services. Multidisciplinary tools were most frequently reported second to diet-specific tools, however, the combination of triaged disciplines varied across studies. Tools were commonly applied to older age cohorts (age mean: 65 years), which may limit generalizability to younger age cohorts. Additionally, nearly 50% of the tools were applied exclusively during treatment; only 20% of tools were universally applied across multiple cancer timepoints (pre, during- and post-treatment). In terms to measurement, most tools measured malnutrition or physical function (60%) and used single cut-off scores to triage (68%), which might not capture or meet the unique physical health needs of each person.

Multiple diet tools exist, where most focus is on malnutrition screening or diagnosis. Tools that reported reach rates greater than 80% included the Malnutrition Universal Screening Tool, 3-Minute Nutrition Screening, Mini Nutritional Assessment, Nutritional Risk Screening 2002, combined assessment of unintentional weight loss, appetite, and serum albumin levels [32], and a nutritional assessment described by Wells et al. [86]. However, these tools all aim to prevent or treat malnutrition. Less consideration is given to weight gain, which is common with chemotherapy and hormone therapy for some cancers, such as prostate and breast cancer [90, 91]. Such tools proposed by Wagner et al. [82]. and Penedo et al. [72] which combine multifaceted diet considerations could be used to facilitate the spectrum of weight management. No diet tools screened for the quality of the diet; emerging evidence suggests reduced overall and cancer-specific mortality [92], and improved health related quality of life in cancer survivors [93]. Inclusion of diet quality screening in diet tools may be of particular importance post-treatment.

This review identified six tools for exercise service triage. When all exercise tool screened impairments were combined, this discipline had the highest overall reach outcomes. However, only two studies that triaged to exercise services provided reach rates [38, 76]. Importantly, there are several physical outcomes that are affected by cancer that exercise can improve, including muscular strength, aerobic fitness and fatigue, among other outcomes [94–96]. However, assessing one isolated outcome at a given time, as was the case with half of the exercise tools, may not account for multiple impairments that will present differently on an inter-individual basis. Furthermore, exercise as a preventative measure to prepare for treatment is emerging within the literature [97, 98], thus screening for likely future impairment alongside current status could be of benefit. This approach is common within the identified diet tools, such as the Nutritional Risk Screening 2002 (NRS-2002) or Malnutrition Universal Screening Tool (MUST). Therefore, the tools that utilised the screening of multiple impairments may appeal to clinicians (ActivOnco, FACT-G, EXCEEDS) [44, 46, 76]. Additionally, the EXCEEDS tool also screened for behaviour change and self-efficacy, an important consideration within healthcare that is less commonly included within triage tools.

Eleven physical rehabilitation tools were identified, which equally captured both physiotherapy and occupational therapy, with speech therapy and cancer physiatry more limited. Interestingly, tools that included a questionnaire or checklist design seemed to have higher reach rates (86–100%) [36, 54, 66] (45-Item Sheffield Profile for Assessment and Referral to Care Questionnaire, Patient-Reported Outcomes Measurement Information System – Cancer Fatigue and Physical Function, Symptom Questionnaire). Another factor that might improve tool completion by the target group for triage to rehabilitation service is the integration into online patient reported management systems (91% reach) [54]. Interestingly, most rehabilitation triage tools were specific to a cancer type, including lung, colorectal, gynaecological, and head and neck, and thus cannot be universally applied.

Over a third of included tools triaged to multidisciplinary services. Although on average three allied health services were included in triage, there was only one multidisciplinary tool that collectively triaged to dietetics, exercise, traditional physiotherapy, and occupational therapy services [77]. To align with models of survivorship [99, 100] and address multifaceted sequalae from cancer and its treatment [101–104], triage tools could include a range of multidisciplinary physical health services (i.e., dietetics, exercise, traditional physiotherapy, occupational therapy, physiatry). However, the availability and provision of all four services will depend on healthcare resources and context. Nevertheless, tools could be designed adaptively to consider the healthcare setting’s resources and healthcare providers. Additionally, services or disciplines that are not available in a specific healthcare setting could be outsourced from external providers via telehealth to allow access to multidisciplinary services.

Nearly a quarter of tools were extracted from a long assessment battery, where tools were used individually to triage to each discipline [31, 32, 41, 42, 63, 64, 67, 71, 79, 81]. Whilst the use of multiple tools is a common approach, the application of a large battery of assessments in real-world settings may face time barriers. Indeed, one study reported a total time of 120 min to complete, with all assessments combined [41]. The provision of supportive care services is already hindered by a lack of time [105, 106] and 83% of surveyed cancer clinicians and allied health professionals (*n* = 58) report that it would be useful for assessments to be no more than 10 min (Rose et al., *unpublished*). Thus, assessment length may impede outcomes such as reach and acceptability. The time limitations posed by multiple singular tools could be overcome by combination tools that create an overall cut-off score to triage to multidisciplinary services, such as those proposed by Capozzi et al. [23], Ray et al. [75], and Young et al. [87]. However, such tools may still pose time concerns (45 min duration [23]), which have been addressed by updated tool versions (Cancer Rehabilitation and Exercise Screening Tool [CREST]) [23]. This tool takes less than 5 min but remains to be validated or implemented [23]. Furthering this, most tools (74%) did not report the time taken which is challenging for clinicians when selecting a tool.

Another consideration is the number of specific decisions a tool can lead to. In this review, over half of tools (68%) led to ‘dichotomous’ triage decisions (i.e., yes or no based on single cut-off scores), rather than stepped care approaches (i.e., multiple levels of interventions for varying impairment severity or risk). The dichotomous approach may not recognise that, while many individuals may need support, not all people following a cancer diagnosis require or may want the same level of support, which depends on their symptom burden, treatments and risk of harm, as well as their goals, preferences and self-efficacy and stage of behaviour change [107]. Two excellent examples that capture the individualisation of allied healthcare provision and triage are that of Schmitz et al. [76] and Wall et al. [83], who utilised the EXCEEDS triage tool [17] (exercise physiology, physiotherapy, occupational therapy; reach = 78%) and a multidisciplinary stepped-care approach (dietetics, speech pathology), respectively. Both tools include a risk rating system, which was used to identify need for intervention type and the priority or triage, respectively.

Importantly, the implementation of a chosen triage tool must be considered to be sure it is suited to and feasible for use within the healthcare context (as well as related barriers and strengths), workflow, and resources [108]. Global implementation barriers of triage tools have been reported in other studies. Notably, patients see the necessity of triage; however, they report limited knowledge of the purpose of assessment [109]. Meanwhile, clinicians often report lack of time, as well as confidence and knowledge of cancer rehabilitation services, which limits triage use [106, 110]. To overcome global and site-specific barriers, an organisation-specific implementation plan, including a defined workflow and ongoing assessment of implementation outcomes, is recommended. Stout et al. proposes an excellent framework that can guide this process [108].

### Limitations

Beyond limited availability of data on application outcomes, studies also reported outcomes inconsistently (e.g., reach, triage). We overcame this through defining and re-calculating these rates (where needed; Table 2*);* however, our calculations were based on available information. Furthering this, as the primary aim was to map available triage tools, studies were included regardless of whether they were conducted under “real world” conditions, thus study designs of non-pragmatic studies could account for higher triage and reach rates. Additionally, over half of the tools (*n* = 49, 63%) originally included or were used in conjunction with non-physical health screening tools which led to services such as nursing, psychological, and medical. This may change the context in which the tool was originally used. Due to a large number of search returns, only title, abstract, and keyword terms were included within the search strategy. This was purposeful – it is likely only articles that specifically used tools for the purpose of physical health triage as a focus of the study would include these terms in the title and abstract, making for a more focussed review. However, it is possible that some relevant studies that included triage details only in the methods might not be included. We also included two interventional terms within the search strategy; 1) triage, and 2) physical rehabilitation. This pragmatic decision limited the large number of search returns. We understand under real-world settings, cancer centres or hospitals have implemented triage tools to inform referrals but may not necessarily publish such assessment tools, processes, or outcomes. This routine clinical practice to peer-review publication gap may have limited search results and overall findings.

## Conclusion

Overall, there are a variety of heterogenous tools used to inform triage to physical health services for people following a cancer diagnosis across dietetics, exercise and physical rehabilitation disciplines. Most tools triaged to dietetics services, applied screening and triage processes during cancer treatment within hospital settings, and measured malnutrition or physical function to inform triage, resulting in single cut-off scores for triage per discipline. Whilst triage tools used to date likely suit the aforementioned contexts and needs (e.g., hospital-based, malnutrition), to progress the field, further triage tools or tool modifications that fill existing gaps are required. Specific gaps include exercise- and rehabilitation-specific tools, potential application across the cancer continuum, varying age cohorts, and multiple cut-off scores for greater individualisation. Furthermore, though dietetic and exercise tools appeared to have the highest triage and reach rates among studies, the lack of reported application outcomes (e.g., reach, acceptability) limits our ability to determine which tools are likely most suitable for context-specific practice, which must also be included in ongoing studies. Nevertheless, our compendium of tools can be used by clinicians to identify triage tools best suited to their setting’s resources, workflow, and physical health support services.

## Supplementary Information

Below is the link to the electronic supplementary material.

Supplementary file1 (DOCX 23 KB)

Supplementary file2 (DOCX 61 KB)

Supplementary file3 (DOCX 28 KB)

Supplementary file4 (DOCX 30 KB)

Supplementary file5 (DOCX 63 KB)

Supplementary file6 (DOCX 37 KB)

Supplementary file7 (DOCX 36 KB)

## Data Availability

No datasets were generated or analysed during the current study.
