# Supplementary material for: Triage tools to inform the prioritisation of physical health services following a diagnosis of cancer: a scoping review
Source: Support Care Cancer. 2025 Aug 6;33(9):760. doi: 10.1007/s00520-025-09816-9 (PMC12328539; doi:10.1007/s00520-025-09816-9)
Supplement: Supplementary file 1 — Supplementary file1 (DOCX 23 KB) [file 520_2025_9816_MOESM1_ESM.docx]

Triage tools to inform the prioritisation of physical health services following a diagnosis of cancer: a scoping review. Supportive Care in Cancer.

Georgia L White, Lauren C Capozzi, Corey Linton, Adrian Wright, Tamara Jones, Hattie H Wright, Kate A Bolam, Elizabeth A Johnston, Briana K Clifford, Keegan Bean, Stephanie Brown, Sarah Kolesaric, Mary A Kennedy, Bryan A Chan, Grace L Rose^1,2^

^1^School of Health, University of the Sunshine Coast, Queensland, Australia

^2^Sunshine Coast Health Institute, Queensland, Australia

E-mail: grose1@usc.edu.au

**Supplementary Table 1a.** Search string for each searched database

| **PUBMED** |
| --- |
| (((((((((((("allied health"[Title/Abstract]) OR ("health care professional*"[Title/Abstract])) OR ("rehab*"[Title/Abstract])) OR ("prehab*"[Title/Abstract])) OR ("supportive care"[Title/Abstract])) OR ("exercise"[Title/Abstract])) OR ("physical activity"[Title/Abstract]) OR("diet*"[Title/Abstract])) OR ("*nutrition*"[Title/Abstract])) AND ((((((((("exercise need*"[Title/Abstract]) OR ("diet* need*"[Title/Abstract]) OR ("rehabilitation need*"[Title/Abstract]) OR ("physical need*"[Title/Abstract]) OR ("needs assessment"[Title/Abstract]) OR (decision*[Title/Abstract]) OR (tool*[Title/Abstract])) OR (triage[Title/Abstract])) OR (referral*[Title/Abstract])) OR (prioritis*[Title/Abstract])) OR (prioritiz*[Title/Abstract])) OR (screening[Title/Abstract]))) AND (((((("cancer*"[Title/Abstract]) OR ("malignan*"[Title/Abstract])) OR ("tumour*"[Title/Abstract])) OR ("tumor*"[Title/Abstract])) OR ("oncolog*"[Title/Abstract])) AND (1995:2024[pdat])) NOT (review[Publication Type])) NOT ("review"[Title]))) NOT ((mice[Title/Abstract]) OR (rat[Title/Abstract]) OR (animal[Title/Abstract])))) |
| **SCOPUS** |
| ( TITLE-ABS ( "allied health" ) OR TITLE-ABS ( "health care professional*" ) OR TITLE-ABS ( "rehab*" ) OR TITLE-ABS ( "prehab*" ) OR TITLE-ABS ( "supportive care" ) OR TITLE-ABS ( "exercise" ) OR TITLE-ABS ( "diet*" ) OR TITLE-ABS ( "nutrition*" ) OR TITLE-ABS ( "physical activity" ) ) AND ( TITLE-ABS ( "decision*" ) OR TITLE-ABS ( "tool*" ) OR TITLE-ABS ( "triage" ) OR TITLE-ABS ( "referral*" ) OR TITLE-ABS ( "prioritis*" ) OR TITLE-ABS ( "prioritiz*" ) OR TITLE-ABS ( "screening" ) OR TITLE-ABS ( "physical need*" ) OR TITLE-ABS ( "needs assessment" ) OR TITLE-ABS ( "diet* need*" ) OR TITLE-ABS ( "exercise need*" ) OR TITLE-ABS ( "rehabilitation need*" ) ) AND ( TITLE-ABS ( "cancer" ) OR TITLE-ABS ( "malignan*" ) OR TITLE-ABS ( "tumour*" ) OR TITLE-ABS ( "tumor*" ) OR TITLE-ABS ( "oncology" ) ) AND NOT DOCTYPE ( re ) AND NOT TITLE ( "review" ) AND NOT TITLE-ABS ( "mice" ) AND NOT TITLE-ABS ( "rat*" ) AND NOT TITLE-ABS ( "animal*" ) AND PUBYEAR > 1994 AND PUBYEAR < 2025 |
| **CINAHL** |
| (TI "allied health" OR TI "health care professional*" OR TI "rehab*" OR TI "prehab*" OR TI "supportive care" OR TI "exercise" OR TI "physical activity" OR TI "diet*" OR TI "nutrition*" OR AB "allied health" OR AB "health care professional*" OR AB "rehab*" OR AB "prehab*" OR AB "supportive care" OR AB "exercise" OR AB "physical activity" OR AB "diet*" OR AB "nutrition*") AND (TI "decision*" OR TI "tool*" OR TI "triage" OR TI "referral*" OR TI "prioritis*" OR TI "prioritiz*" OR TI "screening" OR TI "exercise need*" OR TI "diet* need*" OR TI "rehabilitation need*" OR TI "physical need*" OR TI "needs assessment" OR AB "decision*" OR AB "tool*" OR AB "triage" OR AB "referral*" OR AB "prioritis*" OR AB "prioritiz*" OR AB "screening" OR AB "exercise need*" OR AB "diet* need*" OR AB "rehabilitation need*" OR AB "physical need*" OR AB "needs assessment") AND (TI "cancer" OR TI "malignan*" OR TI "tumour*" OR TI "tumor*" OR TI "oncology" OR AB "cancer" OR AB "malignan*" OR AB "tumour*" OR AB "tumor*" OR AB "oncology") NOT TI (mice or rats or rodents or animals) NOT AB (mice or rats or rodents or animals) NOT TI review NOT PT (review or review of literature review or meta-analysis or systematic review) |

**Supplementary Table 1b.** Detailed inclusion and exclusion criteria

| **INCLUSION CRITERIA** | |
| --- | --- |
| *Population* | - Adults (18+ years) post-cancer diagnosis (any time from point of diagnosis to following completion of treatment) - Where publication includes a mixed cohort, oncology population can be separated from other population groups |
| *Intervention* | - Electronic or physical ‘tool’ that measures physical needs for the purpose of triage or prioritisation   - ‘Tool’ includes a specified assessment or outcome measure that is used to triage to service, OR a triaging system or decision-tree used to prioritise to a service where assessment is used to inform that prioritisation. - Tool was used/ applied to lead to physical allied health care referral, service or similar including:   - Any exercise, physical activity or exercise-centred physical rehabilitation service (e.g., exercise physiology, physiotherapy, occupational therapy, speech pathology, physiatry)   - Any dietary service (e.g., dietetics, nutrition) |
| *Design* | - Randomised Controlled Trials - Quasi-Randomised Controlled Trials - Cohort studies - Pre-post single arm studies - Cross-sectional studies reporting a retrospective model of care |
| *Control* | - Usual care - Control or comparison group receiving a differing intervention - Nil comparison group |
| *Outcome* | - Any outcome relating to the delivery of the screening service or patients |

| **EXCLUSION CRITERIA** | |
| --- | --- |
| *General* | - No full text available - Published in languages other than English |
| *Design* | - Incorrect publication type   - Poster presentation   - Conference proceedings   - Review article   - Single case study |
| *Population* | - Where publication includes a mixed cohort, oncology population cannot be separated from other population groups included |
| *Intervention* | - Data of interest specific to triage tools cannot be adequately extracted - Tool only measures psychological wellbeing [e.g., distress, anxiety, depression] without any other physical symptom measurement - Assessment or outcome used to triage is not specified - Tool was not used to lead to/inform who receives physical rehabilitation service [i.e., only mentions that the tool could be used] |
