# Supplementary material for: Triage tools to inform the prioritisation of physical health services following a diagnosis of cancer: a scoping review
Source: Support Care Cancer. 2025 Aug 6;33(9):760. doi: 10.1007/s00520-025-09816-9 (PMC12328539; doi:10.1007/s00520-025-09816-9)
Supplement: Supplementary file 2 — Supplementary file2 (DOCX 61 KB) [file 520_2025_9816_MOESM2_ESM.docx]

Triage tools to inform the prioritisation of physical health services following a diagnosis of cancer: a scoping review. Supportive Care in Cancer.

Georgia L White, Lauren C Capozzi, Corey Linton, Adrian Wright, Tamara Jones, Hattie H Wright, Kate A Bolam, Elizabeth A Johnston, Briana K Clifford, Keegan Bean, Stephanie Brown, Sarah Kolesaric, Mary A Kennedy, Bryan A Chan, Grace L Rose^1,2^

^1^School of Health, University of the Sunshine Coast, Queensland, Australia

^2^Sunshine Coast Health Institute, Queensland, Australia

E-mail: grose1@usc.edu.au

**Supplementary Table 2.** Summary of included tools by impairment and referred to dietetic services

| Study | Setting | Population | | | | | | Tools | Screening delivered by | Time taken | Application outcomes | | | Triage information | |
| --- | --- | --- | --- | --- | --- | --- | --- | --- | --- | --- | --- | --- | --- | --- | --- |
| Author  Year  Country |  | Sample size (n) | Sex females  n (%) | Age (years)  Mean±SD  where not stated | Cancer type | Cancer stage | Cancer timepoint |  |  | (min) | Acceptability of the tool(s) | Reach | Findings related to patient needs identified | | **HP:** Health professional  **D:** Triage decision design  **R:** Results in  **T:** Triage rate |
| MALNUTRITION RISK | | | | | | | | | | | | | | | |
| 1. Akmansu et al., 2022  Turkey | Cancer clinic | 394 | NR | NR | Mixed | NR | Pre-treatment | NRS-2002 | Oncologist | NR | NR | NR | 34% were at risk of malnutrition | | **HP:** NR  **D:** Cut-off score  **R:** Specific prescription  **T:** 100% |
| 3. Belloumini et al., 2024  Italy | Hospital (public) | 38 | 13 (34) | 66.6±9.5 | Thoracic | II-IV | Pre-treatment | NRS-2002 | Oncologist | 15 | NR | 40% | 18% were at malnutrition risk | | **HP:** Dietitian  **D:** Cut-off score  **R:** Referral  **T:** 100% |
| 10. Chao et al., 2025  Tawain | Hospital (private) | 444 | 109 (25) | NR | Mixed | NR | NR | MUST | Nurse | NR | NR | 84% | 84% were at malnutrition risk | | **HP:** Dietitian  **D:** Cut-off score  **R:** Referral  **T:** 100% |
| 11b. Chapman et al., 2014  USA | Cancer clinic | 211 | NR | Mean: 80.7, range 61-95 | Mixed | NR | Universal | ^MNA | Dietitian | NR (all assessments: 120) | NR | 71% | 62% were at malnutrition risk or malnourished | | **HP:** Dietitian  **D:** Cut-off score  **R:** Specific prescription  **T:** NR |
| 12b. Chebl et al., 2024  USA | Hospital (public) | 50 | NR | 74±NR | NR | NR | During treatment | MNA-SF,  SSA | Nurse | 5 (56 in total) | NR | NR | NR | | **HP:** Dietitian  **D:** Cut-off score  **R:** Referral  **T:** NR (only raw data n=5 referrals) |
| 13. Chen et al., 2012  Singapore | Hospital (public) | 48 | NR | NR | NR | NR | During treatment | 3-MinNS | Nurse | NR | NR | 100% | NR | | **HP:** Dietitian  **D:** Cut-off score  **R:** Referral  **T:** NR |
| 15. Croisier et al., 2022  Australia | Hospital (public) | 104 | 104 (100) | 62±15.6 | Gynaecological | NR | During treatment | MST | Nurse | NR | NR | Baseline = 51.9%  Week 1 = 37.4%  Week 2 = 42.9%  Week 3 =47.6%  Week 4 =70.9%  Week 5 = 30.3%  Week 6 =13.8%  Week 7 = 22.2%  Acute phase 0-6 weeks = 7.6%  Intermediate phase 6 weeks -6 months= 5% | At risk (MST ≥2)  Baseline = 5.8%  Week 1 = 6.1%  Week 2 = 5.5%  Week 3 = 10.7%  Week 4 = 35.4%  Week 5 = 5.3%  Week 6 = 3.4%  Week 7 = 0%  Acute phase 0-6 weeks = 3.3%  Intermediate phase 6weeks-6 months = 1%  28% were at malnutritional risk but were not screened or inaccurately screened with the MST | | **HP:** Dietitian  **D:** Cut-off score  **R:** Referral  **T:** 79.4% |
| 18. Deng et al., 2023  China | Hospital (public) | 108 | 43 (40) | NR | Head and neck | NR | During treatment | NRS-2002 | NR | NR | NR | 100% | 51% score ≥3 | | **HP:** NR  **D:** Cut-off score  **R:** Specific prescription  **T:** 100% |
| 20. Eurich et al., 2022  USA | Cancer clinic | 187 | 187 (100) | Median 57, range NR | Gynaecological | NR | Pre-treatment | MST | Patient care coordinator | NR | NR | 21% | 16% positive malnutrition screen | | **HP:** Dietitian  **D:** Cut-off score  **R:** Referral  **T:** 38% |
| 21. Extermann et al., 2004  USA | Cancer clinic | 11 | 11 (100) | Median 79, range 72-87 | Breast | I-II | During treatment | ^MNA | Dietitian, geriatric oncologist | NR | NR | 66.67% | n=8 nutritional risk | | **HP:** Dietitian  **D:** Stepped care  **R:** Specific prescription  **T:** NR (n=18 nutritional interventions delivered) |
| 30. Jost et al., 2023  Germany | Cancer clinic | 1657 | Period 1: 351 (41) Period 2: 332 (42) | NR | Gastrointestinal | NR | During treatment | PG-SGA | Self-administered | NR | NR | 57.8% | NR | | **HP:** Dietitian  **D:** Cut-off score  **R:** Referral  **T1:** Usual care: 100%, Intervention group: 35.4% |
| 32. Kollar et al., 2022  Hungary | Hospital (public) | 259 | 107 (41) | 68.19±10.7 | Colorectal | NR | Pre-treatment | NRS-2002 | Multiple (surgeon or dietitian) | 5-30 | NR | NR | 62.2% at risk of malnutrition | | **HP:** Dietitian  **D:** Cut-off score  **R:** Specific prescription  **T:** 100% |
| 33. Kufeldt et al., 2018  Germany | Hospital (public) | 840 | 555 (66) | 62.99±12.3 | Mixed | NR | During treatment | NRS-2002 | Nurse | NR | NR | 79.29% | 40.95% at malnutritional risk | | **HP:** NR  **D:** Cut-off score  **R:** Specific prescription  **T:** 97.4% |
| 34. Laursen et al., 2020  USA | Cancer clinic | 317 | 96 (30) | Median 65, range 18-92 | Head and neck | NR | During treatment | abPG_SGA | Self-administered | 5 | NR | 72% | 38% at risk for malnutrition | | **HP:** Dietitian  **D:** Stepped care  **R:** Referral, specific prescription, education  **T:** 36% |
| 36. Levonyak (1) et al., 2021  USA  37. Levonyak (2) et al., 2022  USA | Hospital (public) | 63 | 27 (43) | 58.8±NR | Mixed | I-IV | During treatment | MST | Multiple (nurse and medical oncologist) | NR | NR | NR (increased from 5.1% to 21.8%. 15%-20% range thereafter) | NR | | **HP:** Dietitian  **R:** Referral  **D:** Cut-off score  **T:** NR (raw data: Cycle 1: average  of 22.7 monthly referrals generated from the MST.  Cycle 2: average  of 14.7 monthly referrals generated from the MST.) |
| 40b. Lund et al., 2021 Denmark | Hospital (public) | 142 | 61 (43) | Median 75, range, 70-86.5 | Colorectal | NR | During treatment | ^MNA | Multiple (geriatric specialists with oncology staff) | NR | NR | NR | 75% at risk of malnutrition | | **HP:** Dietitian  **D:** Cut-off score  **R:** Referral and specific prescription (nutritional supplements)  **T1:** 51% |
| 44b. Mohile et al., 2021  USA | University | 718 | 311 (43) | 77.2±5.4 | Mixed | III-IV | During treatment | ^MNA,  low BMI,  > 10% change in weight from 6 months ago | Research staff | NR | NR | 97.95% | Intervention group malnutrition risk: 60%  Control group malnutrition risk: 62% | | **HP:** Dietitian  **D:** Cut-off score  **R:** Referral  **T1:** 44.1% |
| 48. NgWai et al., 2018  Malaysia | Cancer clinic | 739 | NR | NR | NR | NR | During treatment | MST | Nurse | NR | NR | Pre-audit: 6.3%  Post-audit: 79.5% | NR | | **HP:** Dietitian  **D:** Cut-off score  **R:** Referral  **T:** Pre audit: 14.3%, post audit: 15.9% |
| 49b. Paillaud et al., 2022  France | Cancer clinic | 475 | 145 (31) | Median 75.2, IQR 70.3-82.2 | Head and neck | NR | Pre-treatment | ^MNA,  low BMI,  weight loss ≥5% in the last 3 months or ≥10% in the last 6 months | Geriatrician | NR | NR | 26% | Malnourished: 11%  At risk: 33.2%  Normal: 55.8% | | **HP:** Dietitian  **D:** Cut-off score  **R:** Referral and specific prescription  **T1:** 49.1% |
| 51. PérezDoménech et al., 2021  Spain | Hospital (funding type unclear) | 573 | 201 (35) | Mean 59.46, range 16-93 | NR | NR | During treatment | NRS-2002 | NR | NR | NR | NR | Malnourished: 34.38%  At risk: 44.68%  Normal: 20.94% | | **HP:** Dietitian  **D:** Cut-off score  **R:** Referral  **T:** NR |
| 52b. Puts (1) et al., 2023  Canada  53b. Puts (2) et al., 2023  Canada | Hospital (funding type unclear) | 350 | 141 (40) | 75.85±9.8 | Mixed | NR | During treatment | ^Weight loss, appetite,  serum albumin level | Nurse, oncologist, geriatrician | NR | Puts (1) et al., 2023: Completely satisfied or satisfied with their (patient) care received at 6 months: 94.2% in the intervention group compared with 97.6% of the control group (P=0.21). | Puts (1): NR (had an assessment started: 96%)  Puts (2): Completed assessment: 93% | Risk of malnutrition or malnourished: n=162 | | **HP:** Dietitian  **R:** Referral, specific prescription, discussion  **T1:** Referral: 32.3%, dietary advice: 38.51% |
| 59b. Thaker et al., 2021  Australia | Hospital (public) | 1942 | 715 (55)  NR for one site | NR | Mixed | NR | Universal | ^MST,  BMI | Nurse, allied health professional | NR | NR | Site 1: 67%  Site 2: 63% | Site 1:  Malnourished: 22%  Site 2: NR | | **HP:** Dietitian  **D:** Cut-off score  **R:** Referral  **T1:** 18% |
| 60. Trujillo et al., 2021  USA | Cancer clinic | 68119 | NR | NR | Mixed | NR | NR | MST | Nurse or medical assistant | NR | NR | 74% | ≥2: 5% | | **HP:** Dietitian  **D:** Cut-off score  **R:** Referral and  nutrition educational materials  **T:** NR |
| 61b. vanWijk et al., 2021  Netherlands | Hospital (public) | 100 | 49 (49) | Median 72, IQR 66-76 | Mixed | NR | During treatment | ^PG-SGA | Nurse, allied health professionals | NR | NR | NR | Malnourished: 42% | | **HP:** Dietitian  **D:** Cut-off score  **R:** Referral  **T:** 54.8% (16.7% already seeing a dietitian) |
| 62. Wagner et al., 2015  USA | Hospital (private) | 636 | 636 (100) | NR | Gynaecological | NR | NR | #^new items and items adapted from the PG-SGA | Self-administered | 10* | NR | 49.8% | n= 178 patients (33%) endorsed at least one item that generated a message notification to dietitians  Most common reasons for messaging to dietitians included:  - interest in information to gain or lose weight (35%)  - feeling full quickly (14%)  - appetite loss (13%)  - constipation (12%), nausea (11%)  - taste changes (9%)  - fatigue that interferes with maintaining adequate nutrition (9%). | | **HP:** Dietitian  **D:** Cut off, key symptoms reported by patient or patient request consult  **R:** Notification messages  **T1:** 33% |
| 64. Wang et al., 2023  China | Hospital (public) | 526 | 238 (45) | NR | NR | NR | During treatment | NRS-2002 | Doctor, nurse, nutritionist | 2-3 | NR | 100% | Before computerised system: ≥3 = 13%  After computerised system: ≥3 = 32% | | **HP:** Nutritionist  **D:** Cut-off score  **R:** Specific prescription  **T:** Before training: 82.9%, after training: 104.5% |
| 68. Zekri et al., 2014  Saudi Arabia | Hospital (funding type unclear) | 97 | 57 (59) | Median 48, IQR 19-87 | NR | NR | NR | #Nutritional screening tool | Allied health professional | NR | NR | NR | Low: 55%  Moderate: 37%  High: 8% | | **HP:** Dietitian  **D:** Stepped care  **R:** Specific prescription  **T1:** standard formula  oral nutritional support**:** 25%, beyond standard formula  oral nutritional support: 11% |
| CACHEXIA | | | | | | | | | | | | | | | |
| 5. Berry et al., 2018  USA | Hospital (private) | 90 | 48 (53) | Low risk: median 66, range 45-89  Moderate to severe risk: median, range 46-86 | Lung | I-IV | During treatment | A/CS-12 | Self-administered | 3-5 | NR | 81% | Anorexia and cachexia risk: 47% | | **HP:** Dietitian  **D:** Cut-off score  **R:** Referral  **T:** 67.4% |
| COMBINATION | | | | | | | | | | | | | | | |
| 2. Baik et al., 2024  China | Cancer clinic | 59 | 49 (83) | 53.9±12.4 | Mixed | I-IV | During treatment | #^Patient Care Anywhere  (weight change, eating, chewing, or swallowing difficulties) | Self-administered | NR | System usability scale total score: 84.09 | NR | NR | | **HP:** Clinical nutritionist  **D:** Cut-off score  **R:** Discussion  **T:** NR |
| 22. Garcia et al., 2019  USA | Cancer clinic | 3521 | 2398 (69) | 57.15±13.3 | Mixed | NR | NR | #^Supportive Care Needs Assessment Checklist | Self-administered | 40* | NR | 51.5% | "I had the following problems that have kept me from eating enough during the past 2 weeks)  - No problems eating: 71.91%  - Did not feel like eating: 5.16%  - No appetite: 15.16%  - Fatigue n: 12.14%  - Things taste funny: 11.35%  - Feel full quickly: 11.07%  - Nausea: 8.57%  - Dry mouth: 6.26%  - Constipation: 6.12%  - Diarrhea: 6.12%  - Smells bother me: 4.92%  - Pain: 4.85%  - Mouth sores: 3.25%  - Other: 2.93%  - Problems swallowing: 2.60%  - Vomiting: 2.00% | | **HP:** Dietitian  **D:** Checklist  **R:** Specific prescription  **T:** NR |
| 50. Penedo et al., 2022  USA | Cancer clinic | 506 | 506 (100) | Median 59.8, IQR 49.7-67.7 | Gynaecological | NR | NR | #^My Wellness Check (nutritional  needs assessment adapted from NCCN Distress Thermometer Problem Checklist) | Self-administered | 8-10* | NR | 60% | Nutritional needs: 32.9%  - General nutrition concerns: 23.7%  - Wanted information regarding supplements, vitamins, herbs: 21.2%  - Difficulty losing weight or had unintentional weight gain: 16.7% | | **HP:** Dietitian  **D:** Checklist  **R:** Referral or specific prescription  **T:** 86.4% |
| 66. Wells et al., 2008  UK | Hospital (funding type unclear) | 43 | 7 (16) | 65±12 | Head and neck | NR | During treatment | #^Nutritional assessment (incl BMI <20) | Nurse | NR | NR | 88% (phase 2) | NR | | **HP:** Dietitian  **D:** Cut-off score  **R:** Referral  **T:** NR |
| 69. Zullig et al., 2019  USA | Cancer clinic | 44 | 29 (66) | 71.5±6.9 | Mixed | I-IV | Pre-treatment | ^SAOP screener | Nurse or oncologist | 2-3 | NR | 77% | Nutritional needs: 43% | | **HP:** NR  **D:** Cut-off score  **R:** Referral  **T:** 37% |

# authors’ original tool

^ included non-physical health aspects, or was used in conjunction with non-physical health tools (e.g., nursing, psychological, medical)

* time reported included other non-physical health screening tools

3-Minns: 3 Minute Nutrition Screening, b: denotes multiple tools extracted from the same study, BMI: Body Mass Index, Combination: combination of impairments assessed, D: triage decision design, HP: health professional, IQR: interquartile range, MNA: Mini Nutritional Assessment, MNA-SF: Mini Nutritional Assessment Short-Form, MST: Malnutrition Screening Tool, MUST: Malnutrition Universal Screening Tool, NCCN: National Comprehensive Cancer Network, NR: Not Reported, NRS-2002: Nutrition Risk Screening 2002, PG-SGA: Patient-Generated Subjective Global Assessment, R: results in, SAOP: Senior Adult Oncology Program screener, T: the number of people referred/number of people needing to be referred (identified from a positive screening), T: triage rate (the number of people referred/number of people needing to be referred [identified from a positive screening]), T1: where T is NR – triage rate 1 (number of people referred/number of total people screened), UK: United Kingdom,
