# Supplementary material for: Triage tools to inform the prioritisation of physical health services following a diagnosis of cancer: a scoping review
Source: Support Care Cancer. 2025 Aug 6;33(9):760. doi: 10.1007/s00520-025-09816-9 (PMC12328539; doi:10.1007/s00520-025-09816-9)
Supplement: Supplementary file 3 — Supplementary file3 (DOCX 28 KB) [file 520_2025_9816_MOESM3_ESM.docx]

Triage tools to inform the prioritisation of physical health services following a diagnosis of cancer: a scoping review. Supportive Care in Cancer.

Georgia L White, Lauren C Capozzi, Corey Linton, Adrian Wright, Tamara Jones, Hattie H Wright, Kate A Bolam, Elizabeth A Johnston, Briana K Clifford, Keegan Bean, Stephanie Brown, Sarah Kolesaric, Mary A Kennedy, Bryan A Chan, Grace L Rose^1,2^

^1^School of Health, University of the Sunshine Coast, Queensland, Australia

^2^Sunshine Coast Health Institute, Queensland, Australia

E-mail: grose1@usc.edu.au

**Supplementary Table 3.** Summary of included tools by impairment and referred to exercise services

| Study | Setting | Population | | | | | | Tools | Screening delivered by | Time taken | Application outcomes | | | Triage information | |
| --- | --- | --- | --- | --- | --- | --- | --- | --- | --- | --- | --- | --- | --- | --- | --- |
| Author  Year  Country |  | Sample size (n) | Sex females  n (%) | Age (years)  Mean±SD  where not stated | Cancer type | Cancer stage | Cancer timepoint |  |  | (min) | Acceptability of the tool(s) | Reach | Findings related to patient needs identified | | **HP:** Health professional  **D:** Triage design decision  **R:** Results in  **T:** Triage rate |
| PHYSICAL INACTIVITY | | | | | | | | | | | | | | | |
| 7. Brick et al., 2023  USA | Hospital (funding type unclear) | 1174 | 479 (41) | 65.8±12.1 | Mixed | NR | NR | #^My Wellness Check (Moving Through Cancer Oncology  Clinicians' Guide to Referring Patients to Exercise  two-item questionnaire, ECOG) | Multiple (Moving Through Cancer Physical Activity: self-administered. ECOG: oncologist-delivered) | NR | NR | 100% | Physical inactivity: 46% | | **HP:** Cancer physiatrist  **D:** Stepped care  **R:** Referral, education materials  **T:** 31% |
| PHYSICAL FUNCTION | | | | | | | | | | | | | | | |
| 40b. Lund et al., 2021  Denmark | Hospital (public) | 142 | 61 (43) | Median 75, range, 70-86.5 | Colorectal | NR | During treatment | ^Gait speed 10m, handgrip strength | Multiple (geriatric specialists with oncology staff) | NR | NR | NR | 72% performed below cut-off scores in at least one of the physical screenings | | **HP:** Physiotherapist  **D:** Cut off score  **R:** Referral  **T1:** 79% |
| CARDIOVASCULAR CAPACITY | | | | | | | | | | | | | | | |
| 61b. vanWijk et al., 2021  Netherlands | Hospital (public) | 100 | 49 (49) | Median 72, IQR 66-76 | Mixed | NR | During treatment | ^6MWT,  CPET | Nurse, allied health professionals | NR | NR | NR | 6MWT: Low physical fitness or low activity level: 64%  CPET anaerobic threshold≤ 11 mL/kg/min: 52% | | **HP:** NR (Exercise clinical trial – PRIOR study)  **D:** Cut off score  **R:** Referral  **T:** 80% |
| COMBINATION | | | | | | | | | | | | | | | |
| 14. Colombo et al., 2018^1^  USA | Hospital (public) | 30 | 30 (100) | 62.08±12.9 | Breast |  | Post-treatment | FACT-G | Self-administered | 20 | NR | NR | Lack of energy: 24.1%  Being bothered by treatment side effects: 20%  Pain: 10%  I am able to work: 86.2%  I am sleeping well: 50% | | **HP:** Physiotherapist, occupational therapist, physical therapist assistant/physiotherapist for exercise discipline  **D:** Clinical judgement  **R:** Referral  **T1:** Physiotherapy: 35.7%, occupational therapist: 7.1%, exercise program: 17.9%,  home exercise program: 31.1% |
| 16. Dalzell et al., 2017^1^  Canada | Cancer clinic | 75 | 49 (65) | 52±15.5 | Mixed | NR | Universal | #ActivOnco Model of Care (Physical activity levels, clinical judgement relating to physical function, BFI, ESAS, DASH) | Allied health professional | NR | NR | NR | Fatigue: 44.4%  Pain: 32.5%  Dyspnoea 9.8%  Peripheral neuropathy symptoms: 9%  Musculoskeletal issues: 58.6%  Balance/coordination deficits: 12.5%  Neurological/sensory deficits: 12%  Lymphoedema: 7% | | **HP:** Occupational therapist, physiotherapist, “rehabilitation hospital” (HP NR), “home exercise program (HP NR)  **D:** Clinical judgement and cut off score  **R:** Referral  **T1: “**Exercise”:  wellness centre: 55%, home exercise programs: 51%, community centre: 2%  Additional “rehabilitation interventions”:  PT-OT department in hospital: 2%, specialised clinics: 16%,  rehabilitation hospitals: 0.5%,  local community service centres: 4%, ActivoOnco manual mobilisations: 4%, private sector: 1% |
| 56. Schmitz et al., 2024^1^  USA | Cancer clinic | 501 | 215 (43) | 64.96±12.7 | Mixed | I-IV | During treatment | The EXCEEDS triage tool | Self-administered | NR | NR | 78% | Difficulty getting in or out of a vehicle, or using public transportation: n=22, 5.6%  Difficulty walking one block without using a mobility aid like a cane or a walker: n=97, 24.8%  Difficulty moving or reaching with your arms: n=40, 10.2%  Difficulty being without a caregiver or another: n=26, 6.7%  Moderate to high pain that you rate as 7 out of 10 (0 = no pain, 10 = highest pain): n=56, 14.3%  Moderate to high fatigue that you rate as 7 out of 10 (0 = no fatigue, 10 = highest fatigue): n=78, 20%  New or worsening muscle weakness or coordination: n=60, 15.4%    Difficulty with eating or drinking: n=15, 3.87%  Difficulty with memory, multitasking, or thinking n=15, 3.87%    Numbness or loss of sensation in feet or hands: n=38, 9.7%  Dizziness or blurred vision, or feeling lightheaded or disoriented: n=13, 3.3%  None of the above: n=182, 46.6%  Fallen in the past 6 months: n=36, 9.2%  Mild daily fatigue that you rate as 3 to 6 out of 10 (0 = no fatigue, 10 = highest fatigue): n=148, 37.9%  Tingling sensation or loss of feeling in your feet or hands: n=88, 22.5%  Difficulty with memory, multitasking, or thinking: n=42, 10.74%  Dizziness after you stand up from either sitting in a chair or from lying down: n=34, 8.7%  Nausea or frequent vomiting/diarrhea that interferes with daily activities: n=22, 5.6%  Frequent feelings of dehydration: n=8, 2%  Loss or gain of more than 5% body weight: n=25, 6.4%  Lack of voluntary control over urination or defecation (incontinence): n=7, 1.79%  Persistent heaviness or swelling in your arm(s), leg(s), or trunk (lymphedema): n=13, 3.3%  During the past week have you performed 3 or more days of physical activity where your heart beats faster and your breathing is harder than normal for 30 min or more?: Yes n=97, 24.8%  During the past week have you performed 2 or more days of physical activity to increase muscle strength, such as lifting weights?: Yes n=53, 13.6% | | **HP: “**community-based exercise” (HP NR), “supervised exercise (HP NR), physiotherapist, occupational therapist  **D:** stepped care  **R:** Referral  **T1:** Community-based exercise: 27.9%  Clinically supervised exercise: 18.7%  Physiotherapy/occupational therapy: 53.7% |

# authors’ original tool

^ included non-physical health aspects, or was used in conjunction with non-physical health tools (e.g., nursing, psychological, medical)

* time reported included other non-physical health screening tools

^1^ Colombo et al, Schmitz et al and Dalzell et al are included within this table rather than Supplementary Table 5 as the focus of the triaged services was exercise

6-Minute Walk Test, b: denotes multiple tools extracted from the same study, CPET: Cardiopulmonary Exercise Test, D: triage decision design, ECOG: Eastern Cooperative Oncology Group Score, HP: health professionals, IQR: interquartile range, NR: not reported, PA: physical activity, PROMS: patient reported outcome, R: results in, T: triage rate (the number of people referred/number of people needing to be referred [identified from a positive screening]), T1: where T is NR – triage rate 1 (number of people referred/number of total people screened), SD: standard deviation, USA: United States of America
