# Supplementary material for: Triage tools to inform the prioritisation of physical health services following a diagnosis of cancer: a scoping review
Source: Support Care Cancer. 2025 Aug 6;33(9):760. doi: 10.1007/s00520-025-09816-9 (PMC12328539; doi:10.1007/s00520-025-09816-9)
Supplement: Supplementary file 4 — Supplementary file4 (DOCX 30 KB) [file 520_2025_9816_MOESM4_ESM.docx]

Triage tools to inform the prioritisation of physical health services following a diagnosis of cancer: a scoping review. Supportive Care in Cancer.

Georgia L White, Lauren C Capozzi, Corey Linton, Adrian Wright, Tamara Jones, Hattie H Wright, Kate A Bolam, Elizabeth A Johnston, Briana K Clifford, Keegan Bean, Stephanie Brown, Sarah Kolesaric, Mary A Kennedy, Bryan A Chan, Grace L Rose^1,2^

^1^School of Health, University of the Sunshine Coast, Queensland, Australia

^2^Sunshine Coast Health Institute, Queensland, Australia

E-mail: grose1@usc.edu.au

**Supplementary Table 4.** Summary of included tools by impairment and referred to physical rehabilitation services (physiotherapy, or occupational therapy, or speech therapy)

| Study | Setting | Population | | | | | | Tools | Screening delivered by | Time taken | Application outcomes | | | Triage information |
| --- | --- | --- | --- | --- | --- | --- | --- | --- | --- | --- | --- | --- | --- | --- |
| Author  Year  Country |  | Sample size (n) | Sex females  n (%) | Age (years)  Mean±SD  where not stated | Cancer type | Cancer stage | Cancer timepoint |  |  | (min) | Acceptability of the tool(s) | Reach | Findings related to patient needs identified | **HP:** Health professional  **D:** Triage design decision  **R:** Results in  **T:** Triage rate |

| **ADLs and/or IADLs** | | | | | | | | | | | | | | |
| --- | --- | --- | --- | --- | --- | --- | --- | --- | --- | --- | --- | --- | --- | --- |
| **4. Bentley et al., 2013**  England | Hospital (public) | 273 | 105 (38) | 71±10 | Lung | IV | Universal | ^SPARC | Self-administered | NR | NR | 86% | Changes in ability to carry out ADLs: 62%  Losing independence: 59%  Changes in ability to carry out usual household tasks: 56%  Feeling tired: 39% | **HP:** Occupational therapist  **D:** Cut off score  **R:** Referral  **T:** 100% |
| **12b. Chebl et al., 2024**  USA | Hospital (public) | 50 | NR | 74±NR | NR | NR | During treatment | Katz and Lawton ADL/IADL scales | Nurse | 5-15 (56 in total) | NR | NR | NR | **HP:** Occupational therapist  **D:** Cut off score  **R:** Referral  **T:** 0% |
| **29. Jensen et al., 2024**  USA | Community | 58 | 39 (67) | 74.1±8.1 | Multiple myeloma | NR | Universal | ^CARG Geriatric Assessment | NR | NR | Mean satisfaction score with study participation was 4.4 on the 5-  point Likert scale (1- poor experience to 5- best experience | 50% | Physical functioning deficits: 79%  Among these deficits  - Dependence in at least one IADL: 75%  - At least one fall: 61% | **HP:** Physiotherapist  **D:** Cut off score  **R:** Referral  **T:** 45.7% |
| **40b. Lund et al., 2021**  Denmark | Hospital (public) | 142 | 61 (43) | Median 75, range, 70-86.5 | Colorectal | NR | During treatment | ^Katz ADL scale | Multiple (geriatric specialists with oncology staff) | NR | NR | NR | NR | **HP:** Occupational therapist  **D:** Cut off score  **R:** Referral  **T:** 2.8% |
| **PHYSICAL FUNCTION** | | | | | | | | | | | | | | |
| **12b. Chebl et al., 2024**  USA | Hospital (public) | 50 | NR | 74±NR | NR | NR | During treatment | TUG | Nurse | 3-5 | NR | NR | NR | **HP:** Physiotherapist  **D:** Cut off score  **R:** Referral  **T:** 0% |
| **27. Gressel et al., 2019**  USA | Cancer clinic | 336 | 336 (100) | 65±12 | Gynaecological | I-IV | Universal | ^PROMIS-Ca Bank v1.1 – Physical Function, PROMIS-Ca Bank v1.1 – Pain Interference, PROMIS-Ca Bank v1.1 – Fatigue | Self-administered | 5-20* | NR | 91% | Physical function: moderate/severe scores 60%  Pain: moderate/severe scores 36%  Fatigue: moderate/severe scores 28% | **HP:** Physiotherapist  **R:** Referral  **T1:** 3% |
| **58. Soto-Perez-de-Celis et al.,** **2021**  USA | Hospital (public) | 133 | 64 (48) | 60.5±27.3 | Mixed | NR | Pre-treatment | ^FACT-G | Self-administer | NR | NR | NR | Physical wellbeing average:  - Intervention group: 19  - Control group: 18.5 | **HP:** Physiotherapist  **D:** Cut off score  **R:** Specific prescription  **T1:** Intervention group 48.5%, control group: 0% |
| **65. Welford et al., 2023**  UK | Hospital (public) | 153 | 81 (53) | Median 71, range 46-90 | Lung | NR | Universal | CFS | Multiple (any member of the lung cancer multidisciplinary team) | NR | NR | NR | NR | **HP:** Occupational therapist  **D:** Cut off score  **R:** Referral  **T:** NR |
| **FALLS and/or BALANCE** | | | | | | | | | | | | | | |
| **49b. Paillaud et al., 2022**  France | Cancer clinic | 475 | 145 (31) | Median 75.2, IQR 70.3-82.2 | Head and neck | NR | Pre-treatment | ^TUG,  falls within past 6 months,  SLS | Geriatrician | NR | NR | 26% | TUG: >20 sec: 12.4% SLS: <5 sec: 41.8% | **HP:** Physiotherapist  **D:** Cut off score  **R:** Referral  **T1:** 22.2% |
| **EATING, CHEWING, SWALLOWING** | | | | | | | | | | | | | | |
| **42. Miki et al., 2018**  Japan | Cancer clinic | 86 | 20 (23) | Median 78, range 75-90 | Gastric | I-IV | Pre-treatment | #Symptom questionnaire, RSST,  MWST,  VFSS | Rehabilitative physician | NR | NR | 100% | Positive results on any of Symptom questionnaire^#^, RSST, MWST tests: 26.7% | **HP:** Speech therapist  **D:** Cut off score  **R:** Referral  **T:** 86.96% |
| **45. Moroney et al., 2020**  Australia | Hospital (public) | 65 | 15 (23) | 71.3±13.4 | Head and neck | NR | During treatment | #The Pathway (EAT-10, DST) | Multiple (EAT-10: self-administered, DST: dietitian) | 54 | NR | 100% | Dysphagia risk: 27.7% | **HP:** Speech pathologist  **D:** Cut off score  **R:** Referral  **T:** 46.7% |

# authors’ original tool

^ included non-physical health aspects, or was used in conjunction with non-physical health tools (e.g., nursing, psychological, medical)

* time reported included other non-physical health screening tools

ADL: Activities Of Daily Living, b: denotes multiple tools extracted from the same study, CARG: Modified Cancer and Aging Research Group, CFS: Clinical Frailty Scale, D: triage decision design, DST: Dysphagia Screening Tool, EAT-10: Eating Assessment Tool-10, FACT-G: Functional Assessment of Cancer Therapy – General, HP: health professional, IADL: Instrumental Activities of Daily Living, IQR: interquartile range, MWST: Modified Water Swallow Test, NR: not reported, PROMIS-Ca: Patient-Reported Outcomes Measurement Information System – Cancer, R: results in, RSST: Repetitive Saliva Swallowing Test, T: triage rate (the number of people referred/number of people needing to be referred [identified from a positive screening]), T1: where T is NR – triage rate 1 (number of people referred/number of total people screened), TUG, SLS: single leg stance, SPARC: 45-Item Sheffield Profile for Assessment and Referral to Care Questionnaire, TUG: Timed Up and Go, UK: United Kingdom, USA: United States of America, VFSS: Videofluoroscopic Swallowing Study
