# Supplementary material for: Triage tools to inform the prioritisation of physical health services following a diagnosis of cancer: a scoping review
Source: Support Care Cancer. 2025 Aug 6;33(9):760. doi: 10.1007/s00520-025-09816-9 (PMC12328539; doi:10.1007/s00520-025-09816-9)
Supplement: Supplementary file 5 — Supplementary file5 (DOCX 63 KB) [file 520_2025_9816_MOESM5_ESM.docx]

Triage tools to inform the prioritisation of physical health services following a diagnosis of cancer: a scoping review. Supportive Care in Cancer.

Georgia L White, Lauren C Capozzi, Corey Linton, Adrian Wright, Tamara Jones, Hattie H Wright, Kate A Bolam, Elizabeth A Johnston, Briana K Clifford, Keegan Bean, Stephanie Brown, Sarah Kolesaric, Mary A Kennedy, Bryan A Chan, Grace L Rose^1,2^

^1^School of Health, University of the Sunshine Coast, Queensland, Australia

^2^Sunshine Coast Health Institute, Queensland, Australia

E-mail: grose1@usc.edu.au

**Supplementary Table 5.** Summary of included tools by impairment and referred to multidisciplinary services

| Study | Setting | Population | | | | | | Tools | Screening delivered by | Time taken | Application outcomes | | | Triage information | |
| --- | --- | --- | --- | --- | --- | --- | --- | --- | --- | --- | --- | --- | --- | --- | --- |
| Author  Year  Country |  | Sample size (n) | Sex females  n (%) | Age (years)  Mean±SD  where not stated | Cancer type | Cancer stage | Cancer timepoint |  |  | (min) | Acceptability of the tool(s) | Reach | Findings related to patient needs identified | | **HP:** Health professional  **D:** Triage decision design  **R:** Results in  **T:** Triage rate |

| **PHYSICAL FUNCTION** | | | | | | | | | | | | | | |
| --- | --- | --- | --- | --- | --- | --- | --- | --- | --- | --- | --- | --- | --- | --- |
| **6. Breen et al., 2012**  Australia | Hospital (funding type unclear) | 40 | 52 (52) | 66±13 | Mixed | I-IV | During treatment | ^NCCN problem checklist | Self-administered | 9* | Happy to use again: 89%  Helped communicate needs: 84%  Realise their experience/feelings were normal: 97%  Realise help was available when needed: 100%  Helpful to recall information/referrals: 60%  Receiving referrals was useful: 79% | 88% | Fatigue: 63%  Physical: 86% | **HP:** Dietitian, physiotherapist, occupational therapist, speech pathologist  **D:** Checklist  **R:** Discussion, information, referral  **T:** NR for each discipline (88% of needed were discussed, 26% required a referral) |
| **11b. Chapman et al., 2014**  USA | Cancer clinic | 211 | NR | Mean: 80-7, range 61-95 | Mixed | NR | Universal | ^VES-13 (to initially categorise as fit, vulnerable or frail),  Katz and Lawton ADL/IADL scales,  TUG | VES-13: self-administered  Other tools: geriatrician | NR (all assessments: 120) | NR | NR | “Fit” 24%  “Vulnerable” 47%  “Frail” 29%  Other tools: NR | **HP:** Physiotherapist, occupational therapist  **D:** Cut off score  **R:** Specific prescription  **T:** NR |
| **28. Hurria et al., 2007**  USA | Hospital (private) | 245 | 175 (71) | 76±7 | Mixed | NR | NR | ^KPS, falls within the past 6 months, ADLs subscale of MOS Physical Health,  IADLs subscale of OARS, BMI,  % unintentional weight loss in past 6 months | Nurse | NR | Questionnaire was  easy to comprehend: 94%  No upsetting  Questions: 96%  The questionnaire did not omit any important questions: 89%  Satisfied with the questionnaire length: 91% | NR | KPS: >70%: 74%  ≥1 fall in past 6 months: 21%  ADLs: mean: 63  (with 0 indicating completely dependent  and 100 indicating fully functional)  Required some  assistance with IADLs: 49%  Underweight: 20%  Obese: 24%  Unintentional weight loss of more than 5% of total body weight over the previous 6 months: 26% | **HP:** NR (nutrition and “rehabilitation” services)  **D:** Cut off score  **R:** Referral  **T:** Nutrition: 52%, “rehabilitation:” 20% |
| **38b. Li et al., 2021**  USA | Hospital (public) | 605 | 357 (59) | 72.2±5.9 | Mixed | I-IV | During treatment | ^ADL MOS subscale,  IADL OARS subscale (IADL subscale is used to refer to a different rehabilitation discipline that is used for the ADL MOS subscale), TUG,  number of falls in last 6 months, Fulmer SPICES | Multiple (self-administered and allied health professional or research team member) | NR | NR | 100% | ADL MOS subscale: 58.3±30.3  IADL OARS: 12.2±2.5  TUG: 11.3±3.6  ≥1 fall within past 6 months: 19.1% | **HP:** “Exercise” (HP NR), occupational therapist  **D:** Cut off score  **R:** Referral and specific prescription  **T:** NR (only raw data: “functional status” intervention/referrals: intervention group: n=900, standard of care group n=481), |
| **44b. Mohile et al., 2021**  USA | University | 718 | 311 (43) | 77.2±5.4 | Mixed | III-IV | During treatment | ^TUG,  OARS Physical Health,  SPPB,  falls history within past 6 months, Katz and Lawton ADL/IADL scales | Research staff  Self-administered | NR | NR | 98% | 90% in intervention group had impaired physical performance  96%% in control group had impaired physical performance  57% in intervention group and control group had impaired functional status | **HP:** Physiotherapist, occupational therapist, exercise NR  **D:** Cut off score  **R:** Referral, information and specific prescription  **T1: “**Physical performance domain”: physiotherapy referral: 23.6%, occupational therapy referral: 11.1%, exercise information and prescription: 83.4%.  “Functional status domain”: referral to physiotherapy: 27.6%, referral to occupational therapist: 13%, exercise information and prescription: 84.5%. |
| **SARCOPENIA** | | | | | | | | | | | | | | |
| **39. Loeliger et al., 2022**  Australia | Hospital (funding type unclear) | 159 | 70 (44) | Median 61, IQR 49-70 | Mixed | NR | NR | #Sarc Pathway (SARC-CalF, handgrip strength) | Allied health assistant (nutrition assistant) | 15 | Patients: Participant acceptability was deemed high for all  domains of the Theoretic Framework of Acceptability, as indicated by 100% of the responses being slightly agree, agree or  strongly agree for those framed in the positive.  Clinicians:  66.7% of clinicians either strongly agreed or agreed that ‘the introduction of the sarc-pathway into clinical care improves the screening, assessment, diagnosis and intervention of sarcopenia for patients.  Clinician acceptability was deemed high for the Theoretic Framework of Acceptability domain asking ‘fits with my personal values’ (100% of responses being slightly agree, agree or strongly agree. | 99.4% | At risk/probable of sarcopenia: 30.2% | **HP:** Dietitian, physiotherapist (exercise focussed)  **D:** Cut off score  **R:** Referral  **T:** Dietetics: 50% (39.6% already seeing dietitian), physiotherapy: 62.4% (22.9% already see physiotherapist) |
| **NUTRITION** | | | | | | | | | | | | | | |

| 38b. Li et al., 2021  USA | Hospital (public) | 605 | 357 (59) | 72.2±5.9 | Mixed | I-IV | During treatment | ^Fulmer SPICES, % unintentional weight loss in last 6 months,  BMI | Multiple (self-administered and allied health professional or research team member) | NR | NR | 100% | >5% weight loss: 39.3%  BMI: mean 26.6±5.6 | HP: Physiotherapist, occupational therapist, nutritionist  D: Cut off score  R: Referral, specific prescription (nutrition only)  T: NR “nutrition” intervention/referrals: intervention group: n=404, standard of care group: n=210) | |
| --- | --- | --- | --- | --- | --- | --- | --- | --- | --- | --- | --- | --- | --- | --- | --- |
| COMBINATION | | | | | | | | | | | | | | |  |
| 8. Capozzi et al., 2023  Canada | Cancer clinic | 54 | 29 (54) | 51±13.5 | Brain | NR | Universal | ECOG,  KPS, SPPB  medical history, neurological examination | Multiple (physical medicine resident, rehabilitation resident physician, and a clinical exercise physiologist) | 45 | 1) Some participants shared feedback on ways to improve the triage clinic, including  providing additional information on the rationale for the types of assessments chosen  2) All participants (i.e., participants and members of the clinical team) (a) felt satisfied with the triage clinic and (b) valued the triage clinic as part of neuro-oncology care.  3) Some participants spoke of feeling uncertain and nervous in advance of the triage clinic, but yet were ultimately satisfied with how the appointment was conducted. | 94.73% | ECOG 1: 57.1%  ECOG 2: 33.9%  KPS 60-90: 91.1%  Neurological deficits: 92.7%  SPPB: 8.9±3.1 | **HP:** Cancer physiatrist, physiotherapist, occupational therapist, exercise specialist  **D**: Stepped care  **R:** Referral  **T:** Exercise: 100% (all that were eligible were referred). Additional referrals: physiatry: 10.2%, physiotherapy: 10.2%, occupational therapy: 30.61% | |
| 9. Cha et al., 2022  America | Cancer clinic | 1016 | 1014 (99.8) | Mean 58, range 22-88 | Breast | I-III | Post-treatment | #^Survivorship Questionnaire | Advanced practice provider | NR | NR | NR | Nutrition concerns: 66.6%  - Would like to lose weight: 59.2%  - Would like to improve diet: 21.3%  - Would like to gain weight: 1.0%  Pain/swelling: 62.0%  - Pain: 48.4%  - Numbness and tingling: 21.9%  - Swelling: 8.3%  - Trouble moving 22 (2.2%)  Activity: 72.7%  - Fatigue: 51.1%  - Would like to increase physical activity: 46.6%  - Weakness: 13.8%  - Short of breath with activity: 8.3% | **HP:** Dietitian, physiotherapist  **D**: Questionnaire  **R:** Discussion, information provided, referral  **T:** NR (**“**Activity/pain” accepted materials: 98.5%, accepted referral: 3.5%.  “Nutrition” accepted materials: 98.8%, accepted referral: 6.8%) | |
| 17. Danielson et al., 2012  Canada | Cancer clinic | 33 | 20 (70) | Mean 66, range 37-85 | Mixed | NR | NR | #^Multidisciplinary  Screening Form | Allied health professional | NR | Very satisfied with clinic experience: 85%  Would recommend to a patient in a similar situation: 97% | NR | NR | **HP:** Occupational therapist, dietitian  **D:** Cut off score  **R:** Referrals, information package  **T1:** Occupational therapy: 36%  Dietetics: 33% | |
| 19. Dolbeault et al., 2011  France | Cancer clinic | 255 | 234 (92) | Mean 59, range 26-85 | Mixed | NR | Pre-treatment | #^Supportive Care Referral Criteria scale | Nurse | NR | NR | 45% | Physical problems: 69.8% | **HP:** Physiotherapist, “nutrition unit” (HP NR)  **D:** Cut off score  **R:** Discussion and referral  **T:** Nutrition: 0.6%, physiotherapy: 34.6% | |
| 23. Ghazali et al., 2011  UK | Hospital (public) | 466 | 185 (40) | 61.5±11 | Head and neck | NR | Post-treatment | ^PCI | Self-administered | NR | NR | Phase 1: 52%  Phase 2: 61% | NR | **HP:** Dietitian, occupational therapist, physiotherapist, speech therapist  **D:** Checklist  **R:** Discussion and referral  **T1:** Speech therapy: 4.9%, occupational therapy: 0%, dietetics: 0.4%, physiotherapy: 1.6% | |
| 24. Girgis et al., 2009  Australia | Community | 356 | 257 (72) | Mean 57.97, range 33-75 | Mixed | NR | NR | #^Clinician feedback sheet (EORTC QLQ-C30, SCNS- SF34, 10 items from Needs Assessment for Advanced Cancer Patient Questionnaire) | Self-administered | NR | Telephone case worker group participants were more likely to strongly agree that study participation had made discussions with their health care practitioners easier. | 26% | Unmet daily living needs  - Telephone case worker group: n=114  - Oncologist/general practitioner group: n=72  Unmet patient care and support needs  - Telephone case worker group: n=66  - Oncologist/general practitioner group: n=35  Physical symptoms/unmet physical needs  - Telephone case worker group: n=164  - Oncologist/general practitioner group: n=93 | **HP:** Occupational therapist, physiotherapist  **D:** Cut off score  **R:** Discussion and referral  **T:** NR | |
| 25. Girgis et al., 2020  Australia | Hospital (public) | 328 | 195 (60) | Mean 62.4, range 35-86 | Mixed | I-IV | Universal | #^PROMPT-Care 2.0 (ESAS, SCNS- SF34) | Self-administered | NR | NR | 66.4% | Fatigue: 39%  Tiredness: 38.4%  General wellbeing: 32.9% | **HP:** Occupational therapist, physiotherapist, “nutrition and dietetics” (HP NR)  **D:** Cut off score  **R:** Referral, discussion  **T:** NR | |
| 26. Girgis et al., 2022  Australia | Hospital (public) | 48 | 19 (40) | 69±9 | Lung | I-IV | Universal | #^PROMPT-Care Version 3 ePROM (ESAS) | Care coordinators | NR | Main themes generated from staff interviews and surveys, and field notes included: (a) benefits of PROM implementation, including perceived improvements in patient care; (b) how to engage non-English speaking patients; (c) the role of care coordinators and the extent of clinicians’ engagement; and (d) value of extending this model to all cancers. Staff perceived that the PROMs assessments significantly improved their clinical service and increased patient contact. Others thought that using the tool in routine care formalizes their current practice while improving the patient experience (Table 5, section B).  Staff highlighted challenges engaging non-English speaking patients and made suggestions regarding implementing PROMs with these patients and those with low digital literacy. Some improvements in procedures were recommended, including patient onboarding and tailoring assessment frequency. | NR | Tiredness: 64.4%  Fatigue: 61.1% | **HP:** Dietitian, physiotherapist, occupational therapist, “lymphedema” (HP NR)  **D:** Cut off score  **R:** Referral  **T:** Dietetics: 30%, physiotherapy: 30%, occupational therapy: 27.78% | |
| 31. Kenis et al., 2018  Belgium | Hospital (funding type unclear) | 8451 | 4526 (54) | Median, 78.7, range 70-101 | NR | NR | Universal | ^MNA-SF,  fatigue (VAS), pain (VAS),  falls within past 12 months,  G8 screening tool | Nurse | NR | NR | NR | Abnormal G8: 69.9% Other tools: NR | **HP:** Occupational therapist, physiotherapist, dietitian, “fall clinic” (HP NR)  **D:** Cut off score  **R:** Referral  **T1:** Dietitian: baseline: 57%, 3 months post baseline: 73%  Occupational therapy: baseline 7.1%, 3 months post baseline: 9%  Physical therapy: baseline: 11.3%, 3 months post baseline: 14.3% | |
| 35. Lethborg et al., 2014  Australia | Hospital (Unclear) | 25 (only n=20 included within analysis) | (45) | Mean 84, range 74.5-92 | NR | NR | NR | ^Subjective questions are “triggers”  (Dietetics: unintentional weight loss, nutrition impact symptoms, upper gastrointestinal tract obstruction, and/or alternative nutrition therapies,  Occupational therapist: ADLs),  Physiotherapy: falls, mobility, cardiorespiratory fitness  Speech therapy: difficulty eating/drinking, managing saliva, difficulty understanding people or written information, difficulty getting message across, changes in thinking or memory | Multiple (doctors and allied health professionals) | NR | NR | 32.5% | NR | **HP:** Dietetics NR, occupational therapist, physiotherapist, speech therapist  **D:** Questions (clinical judgement)  **R**: Referral  **T1:** Dietetics: Cancer hospital: 50%, general hospital: 50%  Note: more than 1 referral made per patient. Only first n-10 patients that were screened were included within analysis)  Occupational therapy: Cancer hospital: 40%, general hospital: 40% (note only the first n=10 patients screened are included within study analysis)  Physiotherapy: Cancer hospital: 40%, general hospital: 80% (note only the first n=10 patients screened are included within study analysis)  Speech therapy: Cancer hospital: 10%, general hospital: 10% (note only the first n=10 patients screened are included within study analysis) | |
| 41. MacEochagain et al., 2024  UK | Hospital (private) | 244 | 185 (76) | Median 77, IQR 73-82 | Mixed | NR | During treatment | ^SAOP3 | Self-administered | NR | NR | NR | Decrease in appetite: 43.9%,  weight loss of >2.5 kg within past 6  months: 42.2%,  change in food tolerance: 27.5%  Changes in walking: 53.3%,  difficulty with stairs: 34.4%,  falls within past 12 months: 30.3%  Difficulty with continence: 32.4%,  difficulty bathing: 25.5%  difficulty dressing: 8.6% | **HP:** Dietitian, physiotherapist, occupational therapist  **D:** Clinical judgement and cut off score  **R:** Referral  **T:** Physiotherapy: 68.9%,  Occupational therapy: 61.1%,  dietetics: 40.2% | |
| 43. Mikkelsen et al., 2023  Denmark | Hospital (public) | 200 | 200 (100) | Median 53, range 31-81 | Breast | I-III | Post-treatment | #^NARR | Nurse | NR | Satisfaction score: mean 8.9  Agreed timing of the NARR was optimal: 88% | NR | Fatigue: 78%  Neuropathy: 58%  Pain: 11.5%  Lymphedema: 10%  Bowel problems: 2.5% | **HP:** Dietitian, “physical exercise” (HP NR), “lymphedema treatment” (HP NR)  **D:** Clinical judgement  **R:** Referral  **T:** NR (reported as % of referrals aimed [n=45], physical exercise: 48.9%, dietetics 17.9%, lymphedema treatment: 89.9%) | |
| 46. Mortensen et al., 2022  UK | Hospital (public) | 65 | 28 (43) | Mean 65, range 41-82.5 | Head and neck | I-IV | Post-treatment | ^PCI | Self-administered | 12 | NR | 70.65% | Frequently identified concerns at time-point 1:  - Swallowing: 27.9%  - Chewing/eating: 59%  - Activity: 27.9%  Frequently identified concerns at time-point 2:  - Swallowing: 15.4%  - Chewing/eating: 21.1%  - Energy levels: 15.4% | **HP:** Swallowing therapist, speech pathologist, physiotherapist, dietitian, “cancer rehabilitation: (HP NR)  **D:** Checklist  **R:** Discussion, referral  **T:** NR (only raw data provided Swallowing Therapist: CG: referrals n=36, IG: referrals 36  Speech Pathologist:  CG: n=20 referrals, IG: n=36 referrals  Physiotherapist  CG: n=5 referrals, IG n=9 referrals  Dietician:  CG n=1 referrals, IG n=0 referrals  Cancer rehab:  CG n=19 referrals, IG n=11 referrals) | |
| 47. Moshofsky et al., 2022  USA | Hospital (public) | 242 | 100 (41) | 61±14 | Mixed | I-IV | During treatment | #^Psychosocial Needs Screening Checklist | Multiple (nurses, social workers) | NR | 45% of survey respondents reported having more conversation with patients about sensitive topics after the checklist implementation.  55% of providers reported the screening survey helped alleviate the lack of time barrier.  50% of providers felt they made more referrals after implementation. The other 50% believe it made no impact on referrals. | 16.6% | Supportive care needs: (only reported by discipline, not concern)  - Exercise/PT: 26%  - OT: NR | **HP:** Occupational therapist, physiotherapist (physiotherapy /exercise)  **D:** Checklist  **R:** Referral  **T:** Occupational therapy/physiotherapy 43%,  Exercise: NR | |
| 52b. Puts (1) et al., 2023  Canada  53b. Puts (2) et al., 2023  Canada | Hospital (funding type unclear) | 350 | 141 (40) | 75.85±9.8 | Mixed | NR | During treatment | ^SPPB,  falls questions from CARG-TT, OARS IADLs, selection of questions from ESAS,  handgrip strength | Nurse, oncologist, geriatrician | NR | Puts (1) et al., 2023: Completely satisfied or satisfied with their (patient) care received at 6 months: 94.2% in the intervention group compared with 97.6% of the control group (P=0.21).  Puts (2)  Oncologists were satisfied with the implementation of the study, they felt it did not interrupt their clinic flow and they perceived the assessment results as confirming their own clinical impressions while also raising awareness about issues they do not routinely assess themselves. The intervention team members indicated they liked the networking aspect of the study, stating that it improved their communication with oncology teams. In terms of the intervention, they indicated that the assessment was similar to their regular assessment and, therefore, easy to implement. However, they did note that double recordkeeping (documenting the assessment and recommendations in both the study database and in the hospital records), as well as summarising the assessment and recommendations in the letter for the patient, was time consuming. Overall, all intervention team members indicated that they received positive feedback from participants and their families about the monthly nursing phone follow-up calls, particularly during COVID-19. | Puts (1): NR (Had an assessment started: 96%)  Puts (2): Completed assessment: 93% | Falls risk: 29.4%  ORAS IADLs 1 or more IADL impairment: 49.1% | **HP: P**hysiotherapist, occupational therapist  **D:** cut off score  **R:** Referral, specific prescription, discussion  **T:** NR | |
| 54. Qin et al., 2023  USA | Hospital (funding type unclear) | 90 | 14 (16) | 68±8.4 | Mixed | NR | NR | #^CaRe Screen – Cancer rehabilitation electronic screening tool | Oncologist | NR | NR | 2.96% | Impaired endurance: 72.1%  Impaired mobility: 65.6%  Unsteadiness: 41%  Difficult with ADLs: 31.1%  Ability to resume normal activities: 27.9% | **HP:** Physiatrist, physiotherapist, occupational therapist, speech therapist  **D:** checklist  **R:** Referral  **T:** Physiatry: 31%, physiotherapy: 52%, occupational therapy: 27%,  speech therapy: 10% | |
| 55. Ray et al., 2020  Australia | Cancer clinic | 185 | 100 (54) | Median 64, IQR 55-71 | Mixed | NR | Universal | ^MST,  low BMI,  AKPS,  IPAQ-PA guidelines, falls/unsteady,  Needs assistance with self-care,  BFI | Allied health assistant | NR | NR | 85.57% | NR (54% with a positive screen overall) | **HP:** Dietitian, occupational therapist, physiotherapy (exercise focussed)  **D:** stepped care  **R**: Referral  **T:** NR | |
| 57. Soo et al., 2022  Australia | Hospital (funding type unclear) | 154 | 66 (43) | Median 75.5, IQR 72.7–79.9 | Mixed | NR | During treatment | ^TUG,  ECOG,  CHSA CFS, fTRST,  CCI, G8 screening tool,  Modification of Adelaide Tool and CARG-TT, MNA | Oncologist | NR | NR | 96% | TUG: NR  ECOG: ≥2: 20%  CHSA CFS: NR  fTRST: NR  CCI: NR  G8: NR  ADL impairments: 19%  IADL impairments: 54%  (both ADL and IADl from modification of Adelaid Tool and CARG-TT)  MNA: ≤11: 62% | **HP:** Dietitian, “exercise” (HP NR) “rehabilitation”: occupational therapist and physiotherapist  **D:** Clinical judgement  **R:** Referral  **T**: NR | |
| 59b. Thaker et al., 2021  Australia | Hospital (public) | 1942 | 715 (55)  NR for one site | NR | Mixed | NR | Universal | ^Katz and Lawton ADL/IADL scales,  TUG,  falls in past 3 months | Nurse, allied health professional | NR | NR | Site 1: 67%  Site 2: 63% | Site 1:  TUG >12 sec: 23%,  IADLs deficits: 15%  Site 2: NR | **HP:** Physiotherapist, occupational therapist  **R:** Referral  **D:** cut off score  **T1:** Physiotherapy: 22%,  OT: not extracted as referrals included cognitive deficits | |
| 63. Wall et al., 2018  Australia | Hospital (public) | 100 | 20 (20) | 63±12 | Head and neck | I-IV | During treatment | ^#ScreenIT  (Radiotherapy induced side effects, FOIS, PG-SGA, NCCN problems checklist, need for supportive care services#) | Self-administered | 5 | NR | NR | Fatigue: 41%  Eating/drinking: 39%  Sleep: 24%  Pain: 22%  Constipation: 21%  PG-SGA 9+: 58%  Severe side effects: 26%  Moderate side effects: 46% | **HP:** Speech pathologist, dietitian  **D:** Stepped care  **R:** “red flags” generated referrals, “yellow flags” patients are contacted, “green flag” continue monitoring  **T:** NR | |
| 67. Young et al., 2023  Australia | Cancer clinic | 83 | 61 (73) | Mean 59, range 28-82 | Mixed | NR | Universal | ^MST,  AKPS,  IPAQ-PA guidelines, falls/unsteady,  needs assistance with self-care,  BFI | Allied health professional | NR | NR | NR | MST: ≥3: 62.7%,  required occasional assistance: 40.2%  AKPS: >80: 31.6%  BFI: moderate fatigue 58% | **HP:** Dietitian, occupational therapist, physiotherapy (exercise focussed)  **D:** stepped care  **R:** Referral  **T:** NR | |

# authors’ original tool

^ included non-physical health aspects, or was used in conjunction with non-physical health tools (e.g., nursing, psychological, medical)

* time reported included other non-physical health screening tools

ADL: Activities Of Daily Living, AKPS: Australia-Modified Karnofsky Performance Status, b: denotes multiple tools extracted from the same study, BFI: Brief Fatigue Inventory, BMI: Body Mass Index, Combination: combination of impairments assessed, CARG-TT: Cancer and Aging Research Group Chemotherapy Toxicity Tool, CCI: Charlson Comorbidity Index, CG: control group, CHSA CFS: Canadian Study of Health and Aging Clinical Frailty Scale, D: triage decision design, DASH: Disabilities Of the Arm, Shoulder and Hand, ECOG: Eastern Cooperative Oncology Group Score, EORTC QLQ-C30: European Organization for Research and Treatment of Cancer QOL of Life Questionnaire, ESAS: Edmonton Symptom Assessment System, EXCEEDS: Exercise in Cancer Evaluation and Decision Support, FACT-G: Functional Assessment of Cancer Therapy – General, Fulmer SPICES Assessment: Sleep Disorders, Problems with Eating or Feeding, Incontinence, Confusion, Evidence of Falls, Skin Breakdown, fTRST: Flemish Version of the Triage Risk Screening Tool, G8: Geriatric Screening Tool, HP: health professional, IADL: Instrumental Activities of Daily Living, IG: intervention group, IQR: interquartile range, IPAQ: International Physical Activity Questionnaire, KPS: Karnofsky Performance Status, MNA: Mini Nutritional Assessment, MNA-SF: Mini Nutritional Assessment Short-Form, MOS: Medical Outcomes Study, NARR: Needs-Assessment for Rehabilitation, NCCN: National Comprehensive Cancer Network, NR: not reported, OARS: Older Americans Resources and Services, PCI: Patient Concerns Inventory, PG-SGA: Patient-Generated Subjective Global Assessment, R: results in, SAOP-3: Senior Adult Oncology-3, SARC-Calf: Sarc-F Combined with Calf Circumference, SCNS- SF34: Supportive Care Needs Survey- Short Form, SSPPB: Short Performance Physical Battery, T: triage rate (the number of people referred/number of people needing to be referred [identified from a positive screening]), T1: where T is NR – triage rate 1 (number of people referred/number of total people screened), TUG: Timed Up and Go, USA: United States of America, VAS: VAS: Visual Analogue Scale, VES-13: Vulnerable Elders Survey
