# Supplementary material for: Triage tools to inform the prioritisation of physical health services following a diagnosis of cancer: a scoping review
Source: Support Care Cancer. 2025 Aug 6;33(9):760. doi: 10.1007/s00520-025-09816-9 (PMC12328539; doi:10.1007/s00520-025-09816-9)
Supplement: Supplementary file 6 — Supplementary file6 (DOCX 37 KB) [file 520_2025_9816_MOESM6_ESM.docx]

Triage tools to inform the prioritisation of physical health services following a diagnosis of cancer: a scoping review. Supportive Care in Cancer.

Georgia L White, Lauren C Capozzi, Corey Linton, Adrian Wright, Tamara Jones, Hattie H Wright, Kate A Bolam, Elizabeth A Johnston, Briana K Clifford, Keegan Bean, Stephanie Brown, Sarah Kolesaric, Mary A Kennedy, Bryan A Chan, Grace L Rose^1,2^

^1^School of Health, University of the Sunshine Coast, Queensland, Australia

^2^Sunshine Coast Health Institute, Queensland, Australia

E-mail: grose1@usc.edu.au

**Supplementary Table 6.** Quality appraisal of included reports

| Study | Study type | MMAT quality rating | | | | | Quality criteria met | |
| --- | --- | --- | --- | --- | --- | --- | --- | --- |
|  |  | 1 | 2 | 3 | 4 | 5 | |  |
| 1. Akmansu et al., 2022  Turkey | Quantitative descriptive | Y | CT | Y | CT | Y | | 3 (60%) |
| 2. Baik et al., 2024 China | Randomised controlled trial | CT | Y | CT | N | N | | 1 (20%) |
| 3. Belloumini et al., 2024  Italy | Quantitative descriptive | Y | CT | Y | N | Y | | 3 (60%) |
| 4. Bentley et al., 2013  England | Non-randomised trial | CT | Y | Y | N | Y | | 3 (60%) |
| 5. Berry et al., 2018  USA | Non-randomised trial | CT | Y | Y | N | Y | | 3 (60%) |
| 6. Breen et al., 2012  Australia | Non-randomised trial | N | Y | Y | N | Y | | 3 (60%) |
| 7. Brick et al., 2023  USA | Non-randomised trial | CT | Y | N | N | Y | | 2 (40%) |
| 8. Capozzi et al., 2023  Canada | Mixed methods | Y | Y | Y | Y | Y | | 5 (100%) |
| 9. Cha et al., 2022 America | Quantitative descriptive | Y | Y | Y | CT | Y | | 4 (80%) |
| 10. Chao et al., 2025  Tawain | Quantitative descriptive | Y | CT | Y | CT | Y | | 3 (60%) |
| 11. Chapman et al., 2014  USA | Quantitative descriptive | Y | Y | CT | N | CT | | 2 (40%) |
| 12. Chebl et al., 2024  USA | Quantitative descriptive | CT | CT | Y | Y | CT | | 2 (40%) |
| 13. Chen et al., 2012  Singapore | Quantitative descriptive | CT | CT | CT | Y | Y | | 2 (40%) |
| 14. Colombo et al., 2018  USA | Quantitative descriptive | Y | Y | Y | N | Y | | 4 (80%) |
| 15. Croisier et al., 2022  Australia | Quantitative descriptive | Y | Y | CT | CT | Y | | 3 (60%) |
| 16. Dalzell et al., 2017  Canada | Quantitative descriptive | CT | CT | N | N | Y | | 1 (20%) |
| 17. Danielson et al., 2012  Canada | Quantitative descriptive | Y | Y | Y | CT | Y | | 4 (80%) |
| 18. Deng et al., 2023  China | Quantitative descriptive | CT | Y | Y | Y | CT | | 3 (60%) |
| 19. Dolbeault et al., 2011  France | Quantitative descriptive | Y | CT | Y | N | Y | | 3 (60%) |
| 20. Eurich et al., 2022  USA | Quantitative descriptive | Y | CT | Y | N | Y | | 3 (60%) |
| 21. Extermann et al., 2004  USA | Non-randomised trial | Y | Y | N | CT | Y | | 3 (60%) |
| 22. Garcia et al., 2019  USA | Quantitative descriptive | Y | CT | Y | CT | Y | | 3 (60%) |
| 23. Ghazali et al., 2011  UK | Non-randomised trial | Y | Y | CT | CT | Y | | 3 (60%) |
| 24. Girgis et al., 2009  Australia | Randomised controlled trial | Y | Y | Y | CT | N | | 3 (60%) |
| 25. Girgis et al., 2020  Australia | Non-randomised trial | Y | Y | Y | Y | Y | | 5 (100%) |
| 26. Girgis et al., 2022  Australia | Non-randomised trial | Y | Y | Y | CT | Y | | 4 (80%) |
| 27. Gressel et al., 2019  USA | Quantitative descriptive | Y | Y | Y | Y | Y | | 5 (100%) |
| 28. Hurria et al., 2007  USA | Non-randomised trial | CT | Y | CT | CT | Y | | 2 (40%) |
| 29. Jensen et al., 2024  USA | Quantitative descriptive | Y | Y | Y | Y | Y | | 5 (100%) |
| 30. Jost et al., 2023 Germany | Quantitative descriptive | Y | Y | Y | Y | Y | | 5 (100%) |
| 31. Kenis et al., 2018  Belgium | Non-randomised trial | N | Y | Y | CT | Y | | 3 (60%) |
| 32. Kollar et al., 2022  Hungary | Non-randomised trial | Y | Y | Y | CT | Y | | 4 (80%) |
| 33. Kufeldt et al., 2018  Germany | Quantitative descriptive | Y | CT | Y | N | Y | | 3 (60%) |
| 34. Laursen et al., 2020  USA | Quantitative descriptive | Y | Y | Y | CT | Y | | 4 (80%) |
| 35. Lethborg et al., 2014  Australia | Mixed methods | Y | Y | CT | CT | N | | 2 (40%) |
| 36. Levonyak (1) et al., 2021  USA | Quantitative descriptive | Y | Y | Y | Y | Y | | 5 (100%) |
| 37. Levonyak (2) et al., 2022  USA | Quantitative descriptive | Y | Y | Y | Y | Y | | 5 (100%) |
| 38. Li et al., 2022 USA | Randomised controlled trial | Y | Y | Y | CT | Y | | 4 (80%) |
| 39. Loeliger et al., 2022  Australia | Non-randomised trial | Y | Y | Y | CT | Y | | 4 (80%) |
| 40. Lund et al., 2021 Denmark | Randomised controlled trial | CT | Y | Y | Y | Y | | 4 (80%) |
| 41. MacEochagain et al., 2024  UK | Quantitative descriptive | Y | Y | Y | CT | Y | | 4 (80%) |
| 42. Miki et al., 2018  Japan | Non-randomised trial | Y | Y | Y | CT | Y | | 4 (80%) |
| 43. Mikkelsen et al., 2023  Denmark | Mixed methods | Y | Y | Y | Y | Y | | 5 (100%) |
| 44. Mohile et al., 2021  USA | Randomised controlled trial | Y | Y | Y | Y | Y | | 5 (100%) |
| 45. Moroney et al., 2020  Australia | Non-randomised trial | Y | Y | CT | CT | Y | | 3 (60%) |
| 46. Mortensen et al., 2022  UK | Randomised controlled trial | Y | N | Y | Y | N | | 3 (60%) |
| 47. Moshofsky et al., 2022  USA | Quantitative descriptive | Y | Y | Y | N | Y | | 4 (80%) |
| 48. NgWai et al., 2018  Malaysia | Quantitative descriptive | Y | CT | Y | Y | Y | | 4 (80%) |
| 49. Paillaud et al., 2022  France | Randomised controlled trial | Y | Y | Y | N | N | | 3 (60%) |
| 50. Penedo et al., 2022 USA | Quantitative descriptive | Y | CT | Y | Y | Y | | 4 (80%) |
| 51. PérezDoménech et al., 2021  Spain | Non-randomised trial | CT | Y | CT | CT | CT | | 1 (20%) |
| 52. Puts (1) et al., 2023  Canada | Randomised controlled trial | Y | Y | Y | Y | Y | | 5 (100%) |
| 53. Puts (2) et al., 2023  Canada | Mixed methods | Y | N | CT | CT | Y | | 2 (40%) |
| 54. Qin et al., 2023 USA | Non-randomised trial | Y | Y | Y | CT | Y | | 4 (80%) |
| 55. Ray et al., 2020 Australia | Mixed methods | Y | CT | CT | CT | Y | | 2 (40%) |
| 56. Schmitz et al., 2024  USA | Quantitative descriptive | Y | Y | Y | Y | Y | | 5 (100%) |
| 57. Soo et al., 2022 Australia | Randomised controlled trial | Y | Y | Y | Y | Y | | 5 (100%) |
| 58. Soto-Perez-de-Celis et al., 2021  USA | Randomised controlled trial | Y | Y | Y | N | Y | | 4 (80%) |
| 59. Thaker et al., 2021  Australia | Quantitative descriptive | Y | CT | Y | CT | Y | | 3 (60%) |
| 60. Trujillo et al., 2021  USA | Quantitative descriptive | Y | Y | Y | CT | Y | | 4 (80%) |
| 61. vanWijk et al., 2021  Netherlands | Quantitative descriptive | CT | Y | Y | CT | Y | | 3 (60%) |
| 62. Wagner et al., 2015  USA | Quantitative descriptive | Y | Y | Y | CT | Y | | 4 (80%) |
| 63. Wall et al., 2018  Australia | Quantitative descriptive | Y | Y | Y | CT | Y | | 4 (80%) |
| 64. Wang et al., 2023  China | Quantitative descriptive | Y | CT | CT | Y | Y | | 3 (60%) |
| 65. Welford et al., 2023  UK | Quantitative descriptive | Y | Y | Y | CT | Y | | 4 (80%) |
| 66. Wells et al., 2008  UK | Non-randomised trial | Y | Y | Y | N | Y | | 4 (80%) |
| 67. Young et al., 2023  Australia | Quantitative descriptive | Y | CT | Y | N | CT | | 2 (40%) |
| 68. Zekri et al., 2014  Saudi Arabia | Non-randomised trial | CT | N | Y | CT | Y | | 2 (40%) |
| 69. Zullig et al., 2019  USA | Quantitative descriptive | Y | CT | Y | CT | Y | | 3 (60%) |
| Total |  | 54/69 | 49/69 | 54/69 | 20/69 | 59/69 | | 1 (20%): 3  2 (40%): 10  3 (60%): 23  4 (80%): 21  5 (100%): 12 |

MMAT: Mixed Methods Appraisal Tool, Y: yes, N: no, CT: can’t tell

Qualitative:

1. Is the qualitative approach appropriate to answer the research question?
2. Are the qualitative data collection methods adequate to address the research question?
3. Are the findings adequately derived from the data?
4. Is the interpretation of results sufficiently substantiated by data?
5. Is there coherence between qualitative data sources, collection, analysis and interpretation?

Randomized controlled trials:

1. Is randomization appropriately performed?
2. Are the groups comparable at baseline?
3. Are there complete outcome data?
4. Are outcome assessors blinded to the intervention provided?
5. Did the participants adhere to the assigned intervention?

Non-randomized trials:

1. Are the participants representative of the target population?
2. Are measurements appropriate regarding both the outcome and intervention (or exposure)?
3. Are there complete outcome data?
4. Are the confounders accounted for in the design and analysis?
5. During the study period, is the intervention administered (or exposure occurred) as intended?

Quantitative descriptive

1. Is the sampling strategy relevant to address the research question?
2. Is the sample representative of the target population?
3. Are the measurements appropriate?
4. Is the risk of nonresponse bias low?
5. Is the statistical analysis appropriate to answer the research question?

Mixed methods

1. Is there an adequate rationale for using a mixed methods design to address the research question?
2. Are the different components of the study effectively integrated to answer the research question?
3. Are the outputs of the integration of qualitative and quantitative components adequately interpreted?
4. Are divergences and inconsistencies between quantitative and qualitative results adequately addressed?
5. Do the different components of the study adhere to the quality criteria of each tradition of the methods involved?
