# Supplementary material for: Triage tools to inform the prioritisation of physical health services following a diagnosis of cancer: a scoping review
Source: Support Care Cancer. 2025 Aug 6;33(9):760. doi: 10.1007/s00520-025-09816-9 (PMC12328539; doi:10.1007/s00520-025-09816-9)
Supplement: Supplementary file 7 — Supplementary file7 (DOCX 36 KB) [file 520_2025_9816_MOESM7_ESM.docx]

Triage tools to inform the prioritisation of physical health services following a diagnosis of cancer: a scoping review. Supportive Care in Cancer.

Georgia L White, Lauren C Capozzi, Corey Linton, Adrian Wright, Tamara Jones, Hattie H Wright, Kate A Bolam, Elizabeth A Johnston, Briana K Clifford, Keegan Bean, Stephanie Brown, Sarah Kolesaric, Mary A Kennedy, Bryan A Chan, Grace L Rose^1,2^

^1^School of Health, University of the Sunshine Coast, Queensland, Australia

^2^Sunshine Coast Health Institute, Queensland, Australia

E-mail: grose1@usc.edu.au

**Supplementary Table 7.** Triaged disciplines by included study

| Study | Triaged discipline(s) | | | | | |
| --- | --- | --- | --- | --- | --- | --- |
| Author  Year  Country | D/N | Ex | OT | PT | ST | Ph |
| 1. Akmansu et al., 2022  Turkey |  |  |  |  |  |  |
| 2. Baik et al., 2024 China |  |  |  |  |  |  |
| 3. Belloumini et al., 2024  Italy |  |  |  |  |  |  |
| 4. Bentley et al., 2013  England |  |  |  |  |  |  |
| 5. Berry et al., 2018 USA |  |  |  |  |  |  |
| 6. Breen et al., 2012 Australia |  |  |  |  |  |  |
| 7. Brick et al., 2023 USA |  |  |  |  |  |   *exercise focussed |
| 8. Capozzi et al., 2023  Canada |  |  |  |  |  |  |
| 9. Cha et al., 2022 America |  |  |  |  |  |  |
| 10. Chao et al., 2025 Tawain |  |  |  |  |  |  |
| 11b. Chapman et al., 2014  USA  11b. Chapman et al., 2014  USA |  |  |  |  |  |  |
| 12b. Chebl et al., 2024  USA |  |  |  |  |  |  |
| 12b. Chebl et al., 2024  USA |  |  |  |  |  |  |
| 12b. Chebl et al., 2024  USA |  |  |  |  |  |  |
| 14. Colombo et al., 2018  USA |  |  |   *exercise focussed |   *exercise focussed |  |  |
| 15. Croisier et al., 2022  Australia |  |  |  |  |  |  |
| 16. Dalzell et al., 2017  Canada |  |  |   *exercise focussed |   *exercise focussed |  |  |
| 17. Danielson et al., 2012  Canada |  |  |  |  |  |  |
| 18. Deng et al., 2023 China |  |  |  |  |  |  |
| 19. Dolbeault et al., 2011  France |  |  |  |  |  |  |
| 20. Eurich et al., 2022  USA |  |  |  |  |  |  |
| 21. Extermann et al., 2004  USA |  |  |  |  |  |  |
| 22. Garcia et al., 2019  USA |  |  |  |  |  |  |
| 23. Ghazali et al., 2011  UK |  |  |  |  |  |  |
| 24. Girgis et al., 2009  Australia |  |  |  |  |  |  |
| 25. Girgis et al., 2020  Australia |  |  |  |  |  |  |
| 26. Girgis et al., 2022  Australia |  |  |  |  |  |  |
| 27. Gressel et al., 2019  USA |  |  |  |  |  |  |
| 28. Hurria et al., 2007  USA |  |  |   *reported “rehabilitation” |   *reported “rehabilitation” |  |  |
| 29. Jensen et al., 2024  USA |  |  |  |  |  |  |
| 30. Jost et al., 2023 Germany |  |  |  |  |  |  |
| 31. Kenis et al., 2018 Belgium |  |  |  |  |  |  |
| 32. Kollar et al., 2022 Hungary |  |  |  |  |  |  |
| 33. Kufeldt et al., 2018  Germany |  |  |  |  |  |  |
| 34. Laursen et al., 2020  USA |  |  |  |  |  |  |
| 35. Lethborg et al., 2014  Australia |  |  |  |  |  |  |
| 36 & 37. Levonyak (1 & 2) et al., 2021  USA |  |  |  |  |  |  |
| 38b. Li et al., 2022  USA |  |  |  |  |  |  |
| 38b. Li et al., 2022  USA |  |  |  |  |  |  |
| 39. Loeliger et al., 2022  Australia |  |  |  |   *exercise focussed |  |  |
| 40b. Lund et al., 2021  Denmark |  |  |  |  |  |  |
| 40b. Lund et al., 2021  Denmark |  |  |  |  |  |  |
| 40b. Lund et al., 2021  Denmark |  |  |  |   *exercise focussed |  |  |
| 41. MacEochagain et al., 2024  UK |  |  |  |  |  |  |
| 42. Miki et al., 2018 Japan |  |  |  |  |  |  |
| 43. Mikkelsen et al., 2023  Denmark |  |  |  |  |  |  |
| 44b. Mohile et al., 2021  USA |  |  |  |  |  |  |
| 44b. Mohile et al., 2021  USA |  |  |  |  |  |  |
| 45. Moroney et al., 2020  Australia |  |  |  |  |  |  |
| 46. Mortensen et al., 2022  UK |  |  |  |  |  |  |
| 47. Moshofsky et al., 2022  USA |  |  |  |  |  |  |
| 48. NgWai et al., 2018  Malaysia |  |  |  |  |  |  |
| 49b. Paillaud et al., 2022  France |  |  |  |  |  |  |
| 49b. Paillaud et al., 2022  France |  |  |  |  |  |  |
| 50. Penedo et al., 2022 USA |  |  |  |  |  |  |
| 51. PérezDoménech et al., 2021  Spain |  |  |  |  |  |  |
| 52b & 53b. Puts (1 & 2) et al., 2023  Canada |  |  |  |  |  |  |
| 52b & 53b. Puts (1 & 2) et al., 2023 |  |  |  |  |  |  |
| Canada |  |  |  |  |  |  |
| 54. Qin et al., 2023  USA |  |  |  |  |  |  |
| 55. Ray et al., 2020 Australia |  |  |  |   *exercise focussed |  |  |
| 56. Schmitz et al., 2024  USA |  |  |   *exercise focussed |   *exercise focussed |  |  |
| 57. Soo et al., 2022 Australia |  |  |   *reported “rehabilitation programs” |   *reported “rehabilitation programs” |  |  |
| 58. Soto-Perez-de-Celis et al., 2021  USA |  |  |  |  |  |  |
| 59b. Thaker et al., 2021  Australia |  |  |  |  |  |  |
| 59b. Thaker et al., 2021  Australia |  |  |  |  |  |  |
| 60. Trujillo et al., 2021  USA |  |  |  |  |  |  |
| 61b. vanWijk et al., 2021  Netherlands |  |  |  |  |  |  |
| 61b. vanWijk et al., 2021  Netherlands |  |  |  |  |  |  |
| 62. Wagner et al., 2015  USA |  |  |  |  |  |  |
| 63. Wall et al., 2018 Australia |  |  |  |  |  |  |
| 64. Wang et al., 2023 China |  |  |  |  |  |  |
| 65. Welford et al., 2023  UK |  |  |  |  |  |  |
| 66. Wells et al., 2008 UK |  |  |  |  |  |  |
| 67. Young et al., 2023 Australia |  |  |  |   *exercise focussed |  |  |
| 68. Zekri et al., 2014 Saudi Arabia |  |  |  |  |  |  |
| 69. Zullig et al., 2019 USA |  |  |  |  |  |  |

b: denotes multiple tools extracted from the same study, D/N: dietetics/nutrition, E: exercise, OT: occupational therapy, Ph: physiatry, PT: physical therapy, ST: speech therapy
